# Integrating Flow Field Geometries within Porous Electrode Architectures for Enhanced Flow Battery Performance

**DOI:** 10.1002/smll.202511327

**Published:** 2025-10-24

**Authors:** Baichen Liu, Rémy Richard Jacquemond, Vanesa Muñoz‐Perales, Simona Buzzi, Johan Hjelm, Antoni Forner‐Cuenca

**Affiliations:** ^1^ Department of Chemical Engineering and Chemistry Eindhoven University of Technology Eindhoven 5600 MB The Netherlands; ^2^ Department of Energy Conversion and Storage Technical University of Denmark Lyngby DK‐2800 Kgs Denmark; ^3^ Electrochemical Processes Unit IMDEA Energy Avda. Ramón de La Sagra 3 Móstoles 28935 Spain

**Keywords:** electrochemical energy storage, flow field designs, mass transport, non‐solvent induced phase separation, porous electrodes, redox flow batteries

## Abstract

The large‐scale adoption of renewable energy demands efficient and cost‐effective storage solutions, with redox flow batteries (RFBs) emerging as promising candidates for grid‐scale applications. However, their deployment remains constrained by high capital costs, largely driven by the need for advanced porous electrodes that balance high surface area, efficient mass transport, and low‐pressure drop. Compared to conventional, carbon‐fiber‐based porous electrodes, non‐solvent induced phase separation (NIPS) offers a versatile manufacturing approach to tailor electrode microstructures and enhance electrochemical performance, yet optimizing mass transport remains a key challenge. Here, a micro‐patterning strategy is introduced that directly integrates flow field architectures into the electrode structure during NIPS fabrication as a potentially scalable manufacturing approach. Inspired by flow field designs used in fuel cells and flow batteries, we imprint groove and pillar micro‐patterns to enhance in‐plane and through‐plane mass transport. Using symmetric iron flow cells and all‐vanadium full cells, pillar‐patterned electrodes, combined with an interdigitated flow field, are shown to significantly reduce mass transfer resistance and improve electrochemical performance while maintaining a low‐pressure drop. This work presents a simple, scalable, and cost‐effective electrode design strategy to boost RFB power density and advance the economic viability of redox flow battery technology.

## Graphical abstract

The performance of redox flow batteries depends on the interplay of fluid, charge, and mass transport across multiple length scales. This work presents a novel strategy—combining non‐solvent induced phase separation and micromolding—to embed millimeter‐scale flow channels within micrometer‐scale porous electrodes, enhancing electrochemical performance while significantly reducing hydraulic resistance and pumping energy.

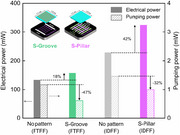

## 1 Introduction

The large‐scale integration of intermittent renewable energy sources into the global energy mix requires the development of cost‐effective energy storage technologies.^[^ ^1^, ^2^ ^]^ Among the various candidates for stationary energy storage, redox flow batteries (RFBs) have gained significant attention due to their long operational lifespan, operational flexibility, and decoupled power and energy capacities.^[^ ^3^, ^4^, ^5^ ^]^ However, despite their promising attributes, the widespread deployment of RFBs remains limited by high capital costs, among other technical challenges, primarily driven by the cost of power – that is, the need for larger stacks to meet high power demands.^[^ ^6^, ^7^ ^]^ One promising path to reducing these costs involves improving power density‐defining components,^[^ ^8^, ^9^ ^]^ particularly the porous electrodes, which govern electrochemical performance and overall energy efficiency.

A typical RFB reactor consists of two porous electrodes separated by a membrane, with flow plates, current collectors, and end plates completing one electrochemical cell in a stack.^[^ ^10^, ^11^ ^]^ The electrode microstructure^[^ ^12^ ^]^ and the design of the adjacent flow fields^[^ ^13^ ^]^ largely determine the electrolyte distribution, mass transport rates, and pressure drop.^[^ ^14^ ^]^ Flow fields, commonly fabricated as engraved patterns on the flow plates, guide the electrolyte through the electrode, ensuring effective utilization of active sites while minimizing pressure losses.^[^ ^15^, ^16^ ^]^ Conventional flow field designs, such as parallel,^[^ ^17^ ^]^ serpentine,^[^ ^18^ ^]^ and interdigitated patterns,^[^ ^19^ ^]^ have been extensively studied. More recently, novel configurations inspired by biological systems or derived through topology optimization have improved electrolyte distribution and reduced transport losses.^[^ ^20^, ^21^, ^22^ ^]^ However, the effectiveness of these flow fields is strongly dependent on the electrode microstructure. A mismatch between the electrode microstructure and the flow field geometry can lead to uneven electrolyte distribution, concentration gradients, and limited active surface utilization, especially when using thicker electrodes.^[^ ^23^, ^24^ ^]^ And, although today these two components are mostly investigated in isolation, it is essential to consider the coupled influence of the flow field and electrode microstructure as integrated components within the reactor design. Looking ahead, there is potential to integrate the flow field and electrode entirely – uniting them into a single, co‐designed structure that optimizes both mass transport and electrochemical performance, while reducing stack complexity and volume.

Porous electrodes in RFBs must simultaneously provide active sites for redox reactions, efficient transport pathways for reactants and products, and minimal hydraulic resistance to allow uniform electrolyte distribution and low pumping power.^[^ ^25^, ^26^ ^]^ Commercial carbon‐fiber‐based electrodes, including carbon paper,^[^ ^27^, ^28^ ^]^ felt,^[^ ^29^, ^30^ ^]^ and cloth,^[^ ^31^, ^32^ ^]^ are commonly used due to their high porosity (e.g., 80–90%),^[^ ^33^ ^]^ good chemical stability, and elevated electronic conductivity. However, these materials exhibit low surface areas (e.g., <10 m^2^ g^−1^),^[^ ^33^ ^]^ limited electrocatalytic activity to inner‐sphere redox couples (e.g. V^2+^/V^3+^, which exhibits a reaction rate constant within 1.2 × 10^−4^ to 1 × 10^−6^ cm s^−1^ on glassy carbon electrode in an acidic electrolyte),^[^ ^34^, ^35^, ^36^, ^37^ ^]^ and microstructures that have not been tailored to sustain convection‐enhanced reactive transport.^[^ ^38^ ^]^ Despite efforts to enhance electrode performance through thermal^[^ ^39^, ^40^ ^]^ and chemical treatments,^[^ ^41^ ^]^ pore etching,^[^ ^42^, ^43^ ^]^ and nanoparticle deposition,^[^ ^44^, ^45^ ^]^ there are fundamental limits related to the intrinsic electrode 3D structure, ultimately limiting the achievable power density.^[^ ^46^ ^]^ Consequently, there is an ongoing effort to develop architected porous electrodes that simultaneously provide high surface area and efficient mass transport pathways. In essence, the challenge is to facilitate mass (convection, diffusion, migration) and fluid transport at multiple length‐scales, which motivates the design of materials with a plurality of geometrical features from nanometer‐ to millimeter‐scale.

Recognizing this fundamental challenge, recent studies have explored the integration of flow fields into the electrode architecture. Strategies such as manual carving,^[^ ^47^, ^48^, ^49^, ^50^, ^51^ ^]^ laser drilling,^[^ ^52^, ^53^ ^]^ and multi‐layer electrode assembly^[^ ^54^, ^55^, ^56^, ^57^, ^58^ ^]^ have been employed to create macroporous pathways (e.g., 2 mm in width), enhancing through‐plane mass transfer and mitigating concentration gradients. While these methods have demonstrated performance improvements, they often rely on labor‐intensive procedures, including multiple additional process steps or yield relatively simple geometric patterns with limited structural control. Recently, topology optimization approaches have demonstrated the potential of micro‐architected electrodes in enhancing transport properties and electrochemical performance.^[^ ^59^, ^60^ ^]^ However, the limited control over pore size and the relatively large and broad pore size distributions of conventional carbon‐fiber electrodes limit the effectiveness of these approaches. Realizing advanced multiscale electrodes requires the precise control of pore size distributions^[^ ^61^ ^]^ and the development of scalable, cost‐effective manufacturing methods compatible with roll‐to‐roll processing.

Non‐solvent induced phase separation (NIPS) offers a promising alternative for manufacturing architected porous electrodes, enabling customizable microstructures with pore sizes ranging from 1 to 100 µm, and potentially broader. Originally developed for membrane fabrication, NIPS enables the production of porous polymeric and carbon‐based materials with tunable pore size distributions and structural gradients.^[^ ^62^, ^63^, ^64^, ^65^ ^]^ Our group recently introduced the NIPS method to manufacture flow battery electrodes.^[^ ^66^, ^67^, ^68^ ^]^ In this process, a polymer solution is cast into a thin film and subsequently immersed in a non‐solvent bath, where the exchange of solvent and non‐solvent induces phase separation. The resulting pore structure is governed by phase separation kinetics, dictated by the interactions between the polymer, solvent, and non‐solvent, as well as the thermodynamic properties of the initial composition.^[^ ^69^ ^]^ Rapid demixing leads to finger‐like macrovoids, which enhance permeability but limit surface area, whereas slower demixing results in sponge‐like structures with high surface area but poorer hydraulic properties. By controlling these parameters, electrodes with tailored porosity and hierarchical architectures can be synthesized. Acknowledging these unique advantages, NIPS‐manufactured electrodes are an excellent material set to introduce multiscale features such as flow channels.

**Figure 1:**
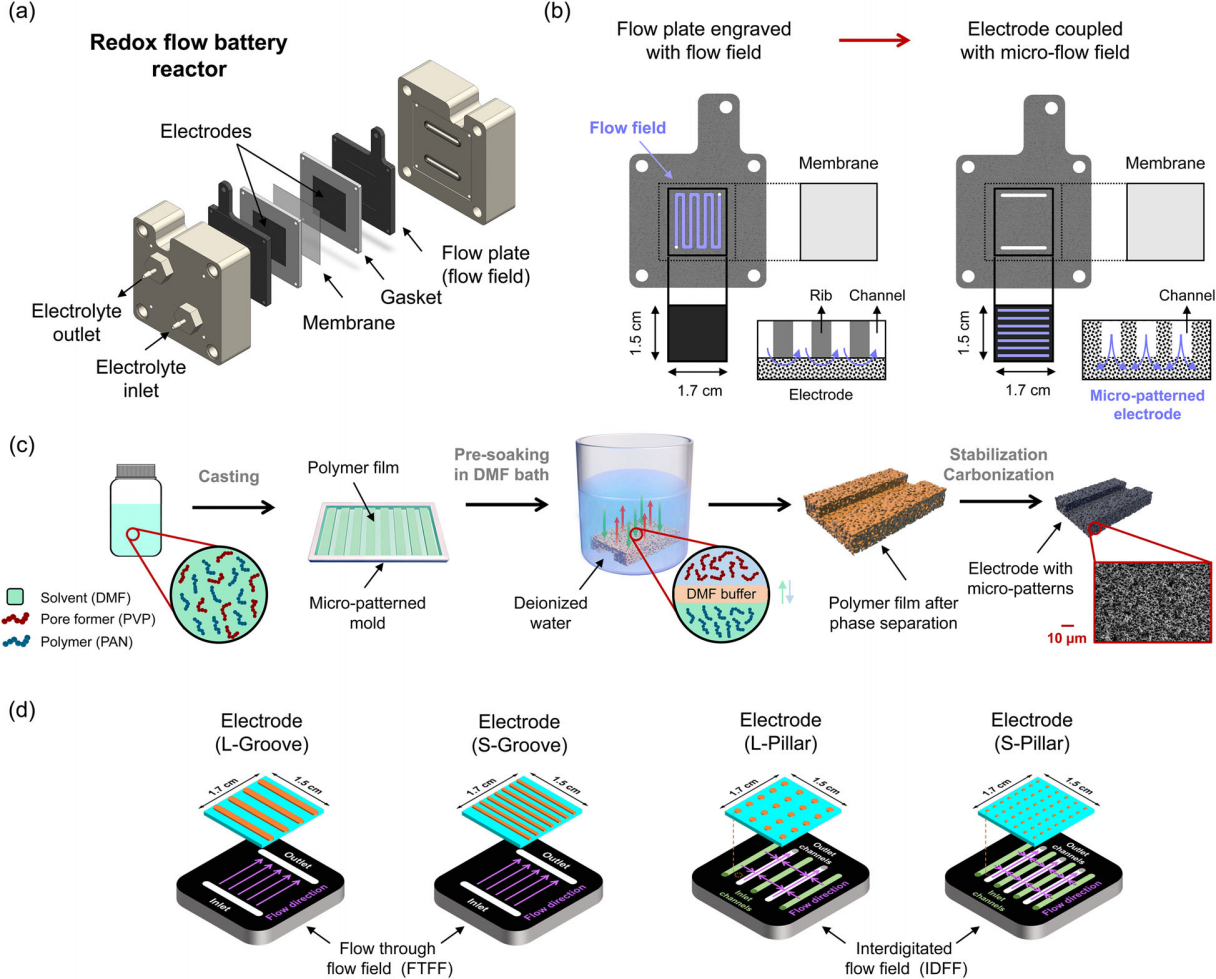
Schematic representations of (a) the redox flow battery reactor design and its main components, b) concept of integrating flow field geometries within the porous electrode, c) fabrication of sponge‐like NIPS electrodes incorporating micro‐patterns, and (d) combination of micro‐patterned NIPS electrodes with conventional flow‐through or interdigitated flow fields.

Micro‐patterns have been introduced via NIPS in tissue engineering and filtration to enhance diffusive flux.^[^ ^70^, ^71^, ^72^ ^]^ In contrast, our work adopts a similar micro‐molding step during NIPS but applies it to fabricate conductive, carbonized porous electrodes for electrochemical flow systems such as RFBs, where the micro‐patterns serve to guide electrolyte flow through the porous structure during cell operation. To address the trade‐off between high electrochemical surface area and poor transport properties, we tailored the concept of micro‐molding specifically for sponge‐like NIPS electrodes. Specifically, we propose a strategy to directly integrate micro‐flow field architectures into porous electrodes during the NIPS manufacturing process (**Figure** **1**a,b). By casting the polymer solution onto patterned molds, microchannels are embedded into the electrode structure, facilitating improved electrolyte distribution and mass transport. We envision that this manufacturing route can produce electrode materials with integrated flow channels (Figure 1c), thereby simplifying the bipolar plate design. The strategy leverages a single‐step molding technique and is potentially compatible with scalable manufacturing methods such as roll‐to‐roll processing. The present work represents a first proof‐of‐concept step toward integrating flow fields directly into electrodes. Further optimization and stack‐level demonstrations will be needed to fully establish this approach for practical applications. In this study, we demonstrate two designs where we target specific mass transport pathways: 1) groove‐patterned electrodes to enhance in‐plane transport and 2) pillar‐patterned electrodes to promote through‐plane flow (Figure 1d).

In this study, we systematically investigate the influence of pattern geometry on hydraulic resistance and electrochemical performance. To assess the effectiveness of these micro‐patterned electrodes, we characterized their structural properties using microscopy and mercury intrusion porosimetry, evaluated permeability via pressure drop measurements, and measured the electrochemically active surface area through electrochemical impedance spectroscopy under non‐faradaic conditions. The electrochemical performance was subsequently evaluated in symmetric iron flow cells and full vanadium RFB cells. Additionally, a 3D continuum model was adapted to simulate electrolyte flow distribution within the patterned electrodes, supporting understanding of the observed performance trends. The proposed strategy demonstrates strong potential for integrating intricate 3D flow fields into electrode designs, enhancing both hydraulic and electrochemical performance. Moreover, this approach may also benefit other RFB chemistry beyond all‐vanadium (e.g., aqueous organic, all‐iron) where there is a need to balance surface area, fluid flow, and mass transport. Furthermore, we envision that adjacent electrochemical devices such as fuel cells^[^ ^61^ ^]^ and electrolysis,^[^ ^73^ ^]^ which rely on multiphase flows at multiple length scales, could benefit from the proposed technology. Overall, our method opens up new opportunities for engineering multiscale structured electrodes across a range of energy conversion and storage technologies.

## 2 Experimental and Computational Methods

### 2.1 Electrode Preparation

Polyacrylonitrile (PAN, Mw ≈150 000 Da, Sigma–Aldrich) and polyvinylpyrrolidone (PVP, Mw ≈ 1 300 000 Da, Sigma–Aldrich) were added in a 100 mL Pyrex bottle and dissolved in N,N‐Dimethylformamide (DMF, ≥99.9%, Sigma–Aldrich) solvent. PAN and PVP were selected as the carbon precursor and pore‐forming agent, respectively.^[^ ^74^ ^]^ The mass ratio of PAN to PVP was selected as 2:3 to obtain an electrode structure with sufficiently high porosity and surface area according to our previous study.^[^ ^66^, ^67^, ^68^ ^]^ To facilitate mixing, a polytetrafluoroethylene (PTFE) magnetic stirring bar was added to the bottle, which was then sealed with parafilm tape. The mixture was stirred on a hot plate magnetic stirrer at 80 °C and 120 rpm for more than 6 h until a homogeneous, clear yellowish solution was formed. The bottle was left on a roller bench at room temperature for over 12 h to ensure complete mixing of the solution and the elimination of gas bubbles.

Aluminum molds (7 × 5 cm^2^ geometry and 1.1 mm thickness) with specific micro‐patterns were designed and manufactured using computer numerical control milling. In this study, we manufactured two different types of micro‐patterns, including groove and pillar patterns. To investigate the influence of the pattern dimensions on the battery performance, we elected to test two different pillar diameters and two groove channel widths. Here, we refer to the patterns with different dimensions as large (L) and small (S). The characteristic dimensions for the large (L) patterns were 1000 µm for both the groove width and the pillar diameter, while the characteristic dimensions for the small (S) patterns were 500 µm. The technical drawings and pictures of the molds can be found in Figure S1 (Supporting Information).

For reproducibility, the molds were positioned side by side in a fumehood to ensure identical environmental conditions during both casting and phase separation. An excess amount of the polymer solution was poured into each mold, and the solution was evenly distributed from one side to the other using a microscopic glass slide (75 × 25 × 1 mm^3^, Epredia), with careful attention to avoid introducing air bubbles on the surface of the polymer film. After the casting process, the phase separation was carried out to facilitate the transition from a polymer‐solvent phase to a nearly pure polymer phase, resulting in the formation of a solid scaffold. The microstructure of the polymer film was finely tuned to yield either a sponge‐like structure or a finger‐shaped macrovoid morphology, with the ultimate structure influenced by the rate of phase separation.^[^ ^66^, ^67^, ^68^ ^]^ In our main study, a sponge‐like structure was achieved by immersing the polymer films (with aluminum molds) in a DMF bath for 5 s. This pre‐soaking step introduced a thin film of DMF solution on the polymer film surface, which moderated the solvent exchange and decelerated the demixing process during phase separation, thereby promoting the formation of a uniform sponge‐like structure. For comparison, in order to obtain the finger‐shaped macrovoid structure (see Figures S2 and S3, Supporting Information), instead of the pre‐soaking process, the polymer film was placed in the fumehood for 15 min after casting, allowing it to absorb moisture on the top surface. In this step, vapor‐induced phase separation was employed to prevent the formation of a dense top layer.^[^ ^75^ ^]^

To initiate the phase separation, a glass container (36.5 × 26.5 × 14.5 cm^3^, Utz Group) filled with 3 L of ultrapure water (18 MΩ, produced from an Elga Purelab Flex water purifier) was prepared as the coagulation bath. After pre‐soaking or vapor‐induced phase separation, the polymer films, with the molds still attached, were carefully placed horizontally in the container to minimize water disturbance. The setup was left undisturbed overnight to allow the solid films to form. The films were then gently detached from the aluminum molds and subsequently immersed in boiling ultrapure water to remove any residual solvents, PVP, and low‐molecular‐weight PAN. After boiling, the membranes were dried using paper towels and then placed between Teflon sheets in a vacuum oven (Memmert 450), set at 80 °C, to dry for >6 h. A ceramic block was placed on top of the membranes to prevent any curling during the drying process.

Following the drying process, the PAN electrodes were transferred to a muffle furnace (Nabertherm, P300) for thermal stabilization, which is an essential step to enhance the mechanical properties of the final electrode material. During stabilization, the PAN electrodes were sandwiched between sheets of alumina paper (Profiltra B.V, thickness: 1 mm) and ceramic plates (13 × 5 × 0.15 cm^3^), with additional weight (≈1200 g) placed on top to ensure uniform compression. The thermal stabilization was conducted in air at 270 °C (ramp rate 2 °C min^−1^) for 1 h and cooled down naturally afterward.

After stabilization, carbonization was performed to form a conductive scaffold. The thermally stabilized PAN electrodes were moved to a tubular furnace (Nabertherm, R 170/1000/13) and heated in a nitrogen environment (LindeGas, grade 5.0). This heating protocol included a gradual temperature increase from room temperature to 850°C (ramp rate 5 °C min^−1^), maintaining this temperature for 45 min. Subsequently, the temperature was increased to 1050 °C (ramp rate 5 °C min^−1^) for an additional 45 min, after which the system was allowed to cool down naturally to room temperature. Finally, the carbonized PAN electrode sheets were placed on a cutting board and cut into smaller sections (1.7 × 1.5 cm^2^) using a sharp razor blade (Home Planet Gear). The whole fabrication process of the sponge‐like NIPS electrodes with micro‐patterns is illustrated in Figure 1c.

In the current battery setup, each NIPS electrode accommodates one of four designs: 4 large grooves (L‐Groove), 7 small grooves (S‐Groove), 20 large pillars (L‐Pillar), or 48 small pillars (S‐Pillar). The groove patterns were oriented perpendicular to the electrolyte flow direction. For the pillar patterns, the dimensions were carefully selected to ensure that each row of pillars was positioned between the inlet and outlet channels in IDFFs. For aligning the large pillars (i.e., L‐Pillar), we adopted IDFF designs with a wider rib width of 2 mm, incorporating 3 inlet channels and 2 outlet channels. For the patterned electrode (S‐Pillar), the normal IDFFs were employed with the rib width of 1 mm, with 4 inlet channels and 3 outlet channels. This arrangement aims to enhance under‐rib convection within the electrode, improving mass transport and overall performance. These electrode‐flow field combinations are illustrated in Figure 1d.

### 2.2 Physicochemical Characterization

Scanning electron microscopy was used to image the electrode structures. A JEOL JSM‐IT100 microscope with an acceleration voltage of 10 kV and a probe current of 12 pA was used. The samples were coated with gold in a JEOL JFC‐2300HR at 90 mA for 60 s.

Mercury intrusion porosimetry (AutoPore IV 9500, Micromeritics) was performed to determine the pore size distribution of the synthesized electrodes. The analysis was conducted using ≈100 mg of electrode material in a 5 cm^3^ volume penetrometer. Pore diameters were calculated assuming cylindrical pores and a mercury‐carbon contact angle of 130° for both advancing and receding interfaces. The bulk porosity was estimated by comparing the mass of the samples before and after complete mercury saturation, assuming full pore infiltration.

X‐ray Photoelectron Spectroscopy (Thermo Scientific K‐alpha) was used to characterize the surface functionalities. The spectrometer was equipped with a monochromatic small‐spot X‐ray source and a 180° double‐focusing hemispherical analyzer with a 128‐channel detector. Spectra were acquired using an aluminum anode (Al Kα = 1486.6 eV) source operating at 72 W and a spot size of 400 µm. Survey scans were conducted at a pass energy of 200 eV, and the region scans used a pass energy of 50 eV. The instrument was maintained at a background pressure of 2 × 10^−8^ mbar, with a measurement pressure of 4 ×1 0^−7^ mbar argon for charge compensation. All acquired spectra were processed and fitted using CasaXPS (Casa Software Ltd).

### 2.3 Pressure Drop Measurement

Pressure drop measurements were performed to quantify the permeability of the electrodes using a custom‐designed flow cell setup (Figure S4, Supporting Information). Two pressure sensors (Stauff SPG‐DIGI‐USB, range: ‐1–16 bar, resolution: 0.1 bar, accuracy: ±0.1 bar) were installed at the inlet and outlet of the half‐cell to measure the pressure differential. Deionized water was used as the working fluid due to its similar density, viscosity, surface tension, and wettability compared to the aqueous electrolyte used in the electrochemical experiments. To isolate the electrode‐induced pressure drop, measurements taken with an empty half cell (same setup without electrode) were subtracted from the recorded values. The pressure drop (Δ*P*) was measured across a range of volumetric flow rates, and the apparent permeability of the electrodes was determined by fitting the data to the non‐linear Darcy–Forchheimer equation. The equation is expressed as follows:

(1) $$\Delta P=\frac{\mu Ql_{e}}{\kappa A_{c}}+\beta \rho {\left(\frac{Q}{A_{c}}\right)}^{2}l_{e}$$

where

 *μ* denotes water viscosity (= 0.89 mPa s at 25 °C);

 *Q* denotes volumetric flow rate;

 *l_e_* denotes the length of the electrode through which the liquid flows;

*A*_c_ denotes electrode cross‐sectional area;

 *β* denotes non‐linear Forchheimer coefficient;

 *ρ* denotes water density (= 997 kg m^−3^ at 25 °C).

### 2.4 Flow Cell Electrochemical Testing

All flow cell experiments were performed in a custom‐designed electrochemical flow reactor (see Figure 1a)^[^ ^66^, ^67^, ^68^ ^]^ with two compartments separated by a Fumasep 950 cation exchange membrane (Fuel Cell Store, dry thickness: 50 µm). Each side of the cell was equipped with a graphite flow field plate (milled from G347B graphite, MWI, Inc.), which ensured uniform electrolyte distribution and functioned as a current collector. Porous electrodes with a geometric area of 2.55 cm^2^ (1.7 × 1.5 cm^2^) were placed in direct contact with the flow field plates. Incompressible polytetrafluorethylene gaskets (ERIKS) were used to seal the flow cell, and the electrodes were positioned within the gasket cavity. The electrodes were compressed to ≈20% of their original thickness by selecting an incompressible gasket with a thickness of 420 µm. The entire assembly was clamped using two polypropylene end plates (McMaster‐Carr), secured by 4 bolts and nuts. After assembly, the cells were tightened to 2.2 N·m using a torque‐controlled wrench. Peristaltic pumps (Cole‐Parmer) were employed to circulate the electrolyte through the cells, with rubber tubing (Masterflex LS‐16) connected to two separate 20 mL electrolyte tanks. Before the operation, the electrolyte was purged with argon for at least 5 min to remove residual air, and it was continuously sparged with humidified argon at room temperature to avoid undesired oxidation.

The electrochemical specific surface area (ECSA) of the porous electrodes was evaluated based on the equivalent double‐layer capacitance (EDLC). For this purpose, electrochemical impedance spectroscopy (EIS) was carried out in a single‐tank battery setup using a 2 m hydrochloric acid solution (HCl, diluted from 37%, Sigma‐Aldrich) as the supporting electrolyte. The impedance was recorded using an amplitude of 10 mV across a frequency range from 200 kHz to 400 mHz, with data points collected at 12 points per frequency decade. An equivalent circuit model was employed to characterize the electrochemical double‐layer charging process. It included an inductor for modeling cable inductance, a resistor for membrane resistance, and a simplified transmission‐line model (Tlm‐Q) to represent a continuously distributed pore network saturated with electrolyte.^[^ ^76^, ^77^, ^78^ ^]^ Under blocking conditions, the surface impedance of the interface between pores and electrolytes can be modelled with a constant phase element (CPE). This equivalent circuit model was established using the Python package impedance.py.^[^ ^79^ ^]^ The best‐fit parameters were obtained from complex non‐linear least squares fitting. The EDLC was estimated through Brug's equation,^[^ ^80^ ^]^ based on the parameters derived from fitting of the experimental EIS data, which is expressed as follows:

(2) $$EDLC={Q_{0}}^{\frac{1}{\alpha }}{\left(\frac{1}{R_{ionic}}\right)}^{1-\frac{1}{\alpha }}$$

where

*Q*_0_ denotes the admittance of the constant phase element;

*R*_ionic_ denotes ionic resistance;

*α* denotes the exponent of the constant phase element;

After obtaining the ECSA, it was normalized by dividing by the volume of the compressed electrode, yielding a volumetric ECSA.

To assess the performance of the novel electrodes in flow batteries, we used two types of flow cell concepts. First, we employed a single‐electrolyte cell configuration to isolate electrode‐related cell overpotentials without secondary effects such as crossover, variations of SoC, or parasitic reactions. Second, we used an all‐vanadium redox flow battery for screening electrodes under a more realistic cell architecture. In the following, we describe the details of these measurements.

The single‐electrolyte cell configuration (Figure S5, Supporting Information) was used to evaluate ohmic, kinetic, and mass transfer overpotential in the micro‐patterned electrodes as it enables to deconvolute of secondary effects (e.g., crossover, parasitic reactions, and variations in the state‐of‐charge over time).^[^ ^81^ ^]^ The electrolytes were prepared by mixing iron(II) chloride tetrahydrate (FeCl_2_·4H_2_O, 98%, Sigma–Aldrich), iron(III) chloride hexahydrate (FeCl_3_·6H_2_O, 97%, Sigma–Aldrich), and HCl in deionized water. The state of charge (SoC) was maintained at 50%, with the total concentration of active species set to 0.5 M in 2 M HCl supporting electrolyte. To ensure the precision of electrolyte flow rates, the pump was calibrated with ultrapure water at each flow rate prior to conducting measurements. For comparative analysis between flow‐through flow‐field (FTFF) and interdigitated flow‐field (IDFF) configurations, four linear electrolyte velocities, i.e., 1, 2, 5, and 10 cm s^−1^, were adopted for both designs. These linear velocities were calculated according to the following equations.

For FTFF:

(3) $$v_{e}=\frac{Q}{t_{e}w_{e}}$$

where

 *v_e_* denotes linear electrolyte velocity;

 *t_e_* denotes compressed electrode thickness;

 *w_e_* denote electrode width.

For IDFF:

(4) $$v_{e}=\frac{Q}{\left(N_{c}-1\right)t_{e}l_{c}}$$

where

*N_c_* denotes the number of channels;

 *l_c_* denotes the length of the flow field channel.

For both FTFF and IDFF configurations, EIS and polarization curves were performed at each linear electrolyte flow velocity to evaluate the overall electrochemical performance. Specifically, EIS was carried out at open circuit voltage, using an amplitude of 10 mV under the frequency range from 200 kHz to 30 mHz with 6 points per frequency decade. Nyquist plots were used to evaluate ohmic, kinetic, and mass‐transfer resistances. Details of the equivalent circuit model are shown in Figure S6 (Supporting Information). Polarization measurements were conducted using potentiostatic holds for 60 s at constant voltage increments of 25 mV from the open circuit voltage (i.e., 0 V) to a cell voltage of 0.6 V. To ensure steady‐state conditions, the median current recorded at each potential step was used as the representative current value. All electrochemical measurements were carried out using Bio‐Logic VMP3 potentiostat.

In the vanadium full‐cell experiments, a two‐tank configuration was employed. The vanadium electrolytes, supplied by OXKEM, were composed of 1.61 m V^3+^ and VO^2+^ (volume ratio of 1:1), 4.05 m SO_4_^2−^, and 0.05 M PO_4_^3−^. The electrolytes were pre‐charged to a SoC of 50% before each test. The electrochemical performance of the NIPS electrodes was compared to the ELAT‐H carbon cloth electrode (Fuel Cell Store). The carbon cloth electrode was thermally activated at 450 °C for 12 h to hydrophilize the electrode and ensure wetting by the electrolyte. For the full‐cell vanadium cycling tests, 12 mL of electrolyte was used on both the positive and negative sides in a two‐reservoir configuration. To precharge the electrolytes, the cell was first charged to 1.7 V and then discharged to 0.8 V at a current density of 200 mA cm^−2^. After precharging, cycling was conducted at current densities ranging from 100 to 400 mA cm^−2^, with 3 cycles performed at each current density. The voltage cut‐off limits were set to 0.8 V (discharge) and 1.7 V (charge). The electrolyte flow rate was maintained at 20 mL min^−1^ throughout all cycling tests.

### 2.5 Cell Continuum Model

Cell continuum models can be leveraged to simulate the influence of reactor design on the convoluted physicochemical phenomena in electrochemical flow cells at low computational cost by neglecting the non‐uniform microstructural effects of porous electrodes. In this context, the unimodal pore size distribution of the sponge‐like NIPS electrodes provides an opportunity to assume the electrode material as a homogeneous domain defined by effective properties (e.g., overall electrode porosity). Because of the macroscopic assumptions, cell continuum models are primarily used to gain qualitative insight into the influence of cell geometry (e.g., flow field design, electrode dimensions), flow configuration, and operating conditions on property profiles (e.g., concentration, velocity) and subsequent performance outputs (e.g., pressure drop).^[^ ^82^, ^83^, ^84^ ^]^ Because of the symmetry of the single‐electrolyte cell (same electrolyte in both half‐cells), we modeled a 3D half‐compartment of the flow cell without including complex phenomena such as crossover. The model used was developed in our previous work, where a detailed description can be found in ref. [85].

The 3D half‐cell model first solved the fluid dynamics through Navier‐Stokes and Brinkman equations in the flow channels and porous electrodes, respectively, to calculate the pressure field from which the local velocity values are obtained. The velocity field was coupled to the mass transport of species through the Nernst‐Planck equation, which captures convection, diffusion, and migration of species. Subsequently, mass and charge transport were bidirectionally coupled through the Butler‐Volmer equation to obtain the reaction source, potential fields, and species concentration. We performed the finite element simulations in COMSOL Multiphysics® in an AMD Ryzen 9 3900 × 12‐Core Processor 3.79 GHz CPU supported by 64 GB of RAM. The establishment of the cell continuum model, including equations, boundary conditions, and geometrical domain, is further described in our previous work^[^ ^85^ ^]^ and Figure S7 (Supporting Information). Some key parameters used in this model were modified based on the experimental measurements, as shown in Table S1 (Supporting Information). The model was validated in our previous study,^[^ ^85^ ^]^ nevertheless, we further validated the model results for the reactor configurations used in this work. The current model was employed for a qualitative analysis of flow and velocity distributions, aiming to identify the factors contributing to performance enhancement. To validate this model, the simulated pressure drop was compared to the experimental data for the no‐pattern NIPS electrodes (Figure S8, Supporting Information). Precisely representing the complex NIPS microstructure, especially in groove and pillar regions, would require a detailed pore‐scale model capable of capturing local gradients, which is beyond the scope of the present study. Here, we focus on a macro‐homogeneous approximation to describe bulk transport behavior qualitatively.

## 3 Results and Discussion

### 3.1 Micro‐Patterned Electrode Morphology

**Figure 2:**
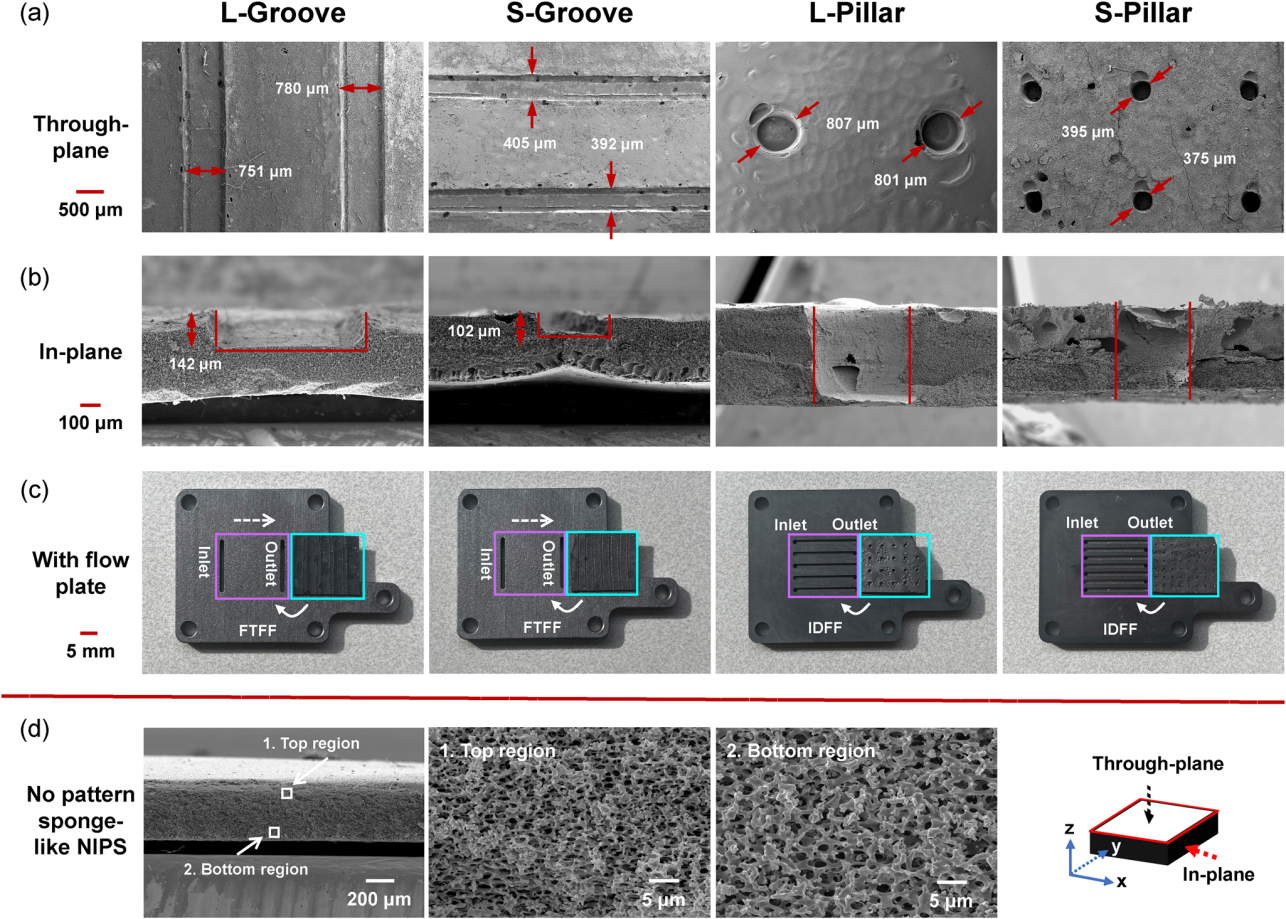
Microstructural characteristics of micro‐patterned electrodes: a) through‐plane and (b) in‐plane SEM images of the sponge‐like NIPS electrodes with different micro‐patterned designs. The through‐plane views are from the bottom side of the electrodes, while the in‐plane views are from the cross‐section. c) Optical photographs of the electrodes with a combination of the flow plates. d) SEM images of the sponge‐like NIPS electrodes without micro‐patterns under different magnifications.

We began by examining the electrode morphology using scanning electron microscopy (SEM). **Figure** **2**a,b contains SEM images of the carbonized, sponge‐like NIPS electrodes with integrated micro‐patterns. The top‐view and cross‐sections confirm the successful incorporation of both groove and pillar micro‐patterns into the electrode structure. A noticeable dimensional shrinkage is observed after carbonization, with distinct differences between the bulk and micro‐patterned regions. Specifically, the shrinkage ratios within the groove and pillar areas range from 20% to 25%, whereas the regions between adjacent grooves or pillars exhibit significantly higher shrinkage, approaching 50%. Although the shrinkage is non‐uniform in the patterned and bulk regions, it is reproducible across batches, making it feasible to pre‐compensate for dimensional changes during mold design. Detailed data are presented in Table S2 (Supporting Information). This differential shrinkage induces deformation along the borders of the micro‐patterned regions, particularly in the groove areas, where the electrode thickness decreases, and the groove walls extend outward. These shrinkage effects should be carefully considered when designing the molds to maintain the structural integrity and mechanical stability of the NIPS electrodes after carbonization.

As shown in Figures 1d and 2c, we combine the groove‐patterned and pillar‐patterned NIPS electrodes with FTFFs and IDFFs, respectively. While FTFF designs are known to efficiently facilitate axial transport of the electrolyte from the inlet to the outlet, they are not effective in achieving uniform lateral distribution.^[^ ^86^ ^]^ We hypothesize that incorporating groove patterns may mitigate this limitation by enhancing in‐plane electrolyte distribution. For IDFF designs, although they inherently promote lateral distribution,^[^ ^87^ ^]^ we expect that the introduction of pillar structures will reduce the pressure drop, thereby enabling deeper through‐plane electrolyte penetration and more uniform distribution throughout the electrode thickness. We posit that strategically aligning electrode microstructures with flow field designs on flow plates could optimize electrolyte transport across multiple spatial dimensions at the microstructural level. We envision that further optimization of pattern geometry within the electrode (e.g., channel spacing, pillar density, aspect ratios) can be realized with the support of computational methods (i.e., topology optimization), but this is beyond the scope of the present study.

For comparison, Figure 2d presents cross‐sectional SEM images of the sponge‐like NIPS electrodes without micro‐patterns (referred to as “no pattern”) at different magnifications. These electrodes exhibit a characteristic sponge‐like microstructure with smaller pores compared to the finger‐shaped NIPS electrodes (see Figure S2, Supporting Information). A slight pore size gradient is observed along the through‐plane direction, with larger pores near the bottom (average diameter: 2.4 µm, measured from SEM images) and smaller pores near the top (average diameter: 1.2 µm, measured from SEM images), as measured from cross‐sectional SEM images. Despite this gradient, mercury intrusion porosimetry measurements reveal a relatively narrow pore size distribution, with a predominant peak ≈1.5 µm (see Figure S3, Supporting Information).

In addition, water contact angle measurements confirm that the surface of the no‐pattern and micro‐patterned sponge‐like NIPS electrodes is hydrophilic (see Figure S9, Supporting Information). Specifically, the no‐pattern and patterned electrodes (S‐Groove and S‐Pillar) show contact angles of 65±2°, 60±1°, and 61±1°, respectively. The incorporation of various micro‐patterns shows minimal impact on the apparent water contact angles. Compression stress‐strain tests show that all of the micro‐patterned electrode maintains mechanical stability under flow battery assembly conditions (see Figure S10, Supporting Information). In the following sections, we compare the hydraulic and electrochemical performance of the micro‐patterned NIPS electrodes with the no‐pattern electrodes to assess the performance enhancements enabled by the integrated micro‐pattern features.

### 3.2 Electrode Permeability and Available Surface Area

**Figure 3:**
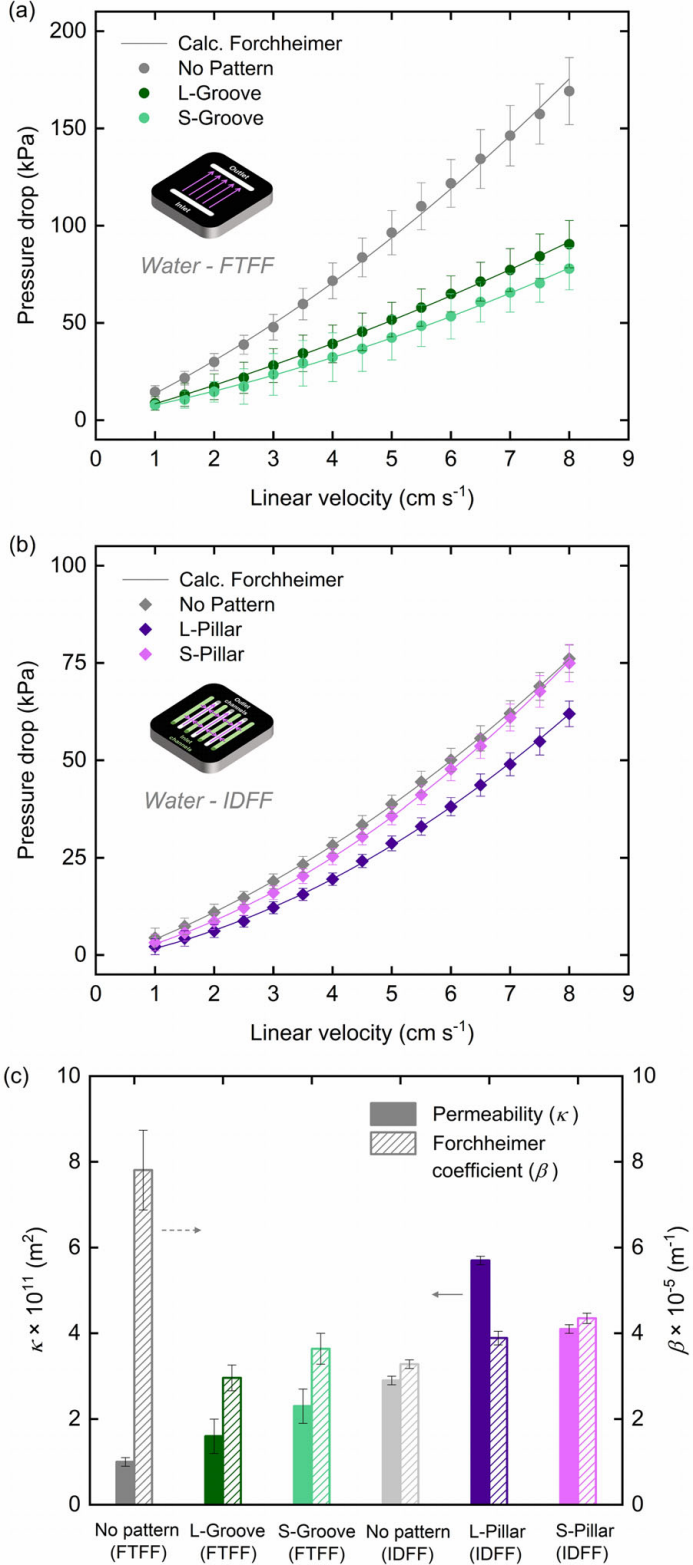
Pressure drops measurements (dots) versus linear electrolyte velocity in the sponge‐like NIPS electrodes for a) micro‐groove patterns in FTFF and b) micro‐pillar patterns in IDFF in a single‐cell, together with the non‐linear Darcy‐Forchheimer fittings (lines). The apparent permeability (*κ*) and Forchheimer coefficient (*β*) results are summarized in (c). The error bars correspond to a standard deviation (*n* = 2).

The pumping power required to sustain the electrolyte flow through the electrochemical reactor impacts the overall energy efficiency of the system. By incorporating micro‐patterns into the NIPS electrodes, the electrolyte penetration pathways within the electrode structure can be tailored to mitigate pressure losses. To assess the hydraulic resistance in the electrochemical cell, the pressure drop of each electrode is measured with the selected flow fields (i.e., groove patterns with FTFF and pillar patterns with IDFF) using water as the working fluid. The pressure drops results for FTFF and IDFF are shown in **Figure** **3**a,b, respectively, and the fitted apparent permeability results are summarized in Figure 3c. The pressure drops results shown in Figure 3 represent the net contribution from the electrode, calculated by subtracting the baseline pressure drop of the empty cell (≈7% of the total). Raw data for both empty and full cell configurations are provided in Figure S11 (Supporting Information).

The baseline NIPS (no pattern) electrode, when combined with the FTFF configuration, exhibits the highest pressure drop, with a measured permeability of ≈1.0 × 10^−11^ m^2^. This permeability is lower than that of commercial carbon‐fiber Freudenberg H23 paper electrodes (3.8 × 10^−11^ m^2^ at 20% compression,^[^ ^88^ ^]^ which is to be expected given the smaller pore diameter (≈1.5 µm, see Figure S3, Supporting Information). Introducing groove patterns perpendicular to the flow direction significantly reduces the pressure drop. For example, the pressure drop decreases from 96.7 kPa in the no patterned electrode to 46.8 kPa in the patterned electrode (S‐Groove) at an electrolyte velocity of 5 cm s^−1^. This reduction is attributed to the partial removal of electrode material to create flow pathways, leading to a 9.1% and 7.3% decrease in volume for the patterned electrodes (L‐Groove and S‐Groove), respectively (see Table S3, Supporting Information). In the FTFF configuration, the electrolyte is forced to traverse shorter distances as it encounters each groove channel, where the local flow resistance is minimized. Additionally, the no‐pattern electrodes exhibit non‐linear behavior in the pressure drop measurements at higher flow velocities, as indicated by a Forchheimer coefficient of 7.8 × 10^5^. In contrast, this non‐linear behavior is less pronounced in the micro‐patterned electrodes, with *β* values of 3.0 × 10^5^ and 3.6 × 10^5^ for the patterned electrodes (L‐Groove and S‐Groove), respectively. A summary of the measured permeability and Forchheimer coefficients for all sponge‐like NIPS electrodes is provided in Table S4 (Supporting Information). Notably, the permeability of the sponge‐like no‐pattern NIPS electrodes is approximately one‐third that of the finger‐shaped electrodes under the same flow field conditions, consistent with previous findings.^[^ ^66^, ^67^, ^68^ ^]^ The detailed results are shown in Table S5 (Supporting Information).

When the no‐pattern NIPS electrode is paired with IDFFs, the pressure drop observed is lower than that with FTFFs at the same linear flow velocities, as the electrolyte travels shorter distances within the porous electrode.^[^ ^19^ ^]^ Incorporating pillar patterns into the electrodes leads to a further reduction in pressure losses, although this effect is less pronounced than with the groove designs in FTFF configurations. Among the pillar patterns, the L‐Pillar design exhibits a more noticeable decrease in pressure drop than the S‐Pillar, with the apparent permeability increasing by ≈90% and 40%, respectively, compared to the no‐pattern electrode. Additionally, the electrode with pillar patterns under IDFF exhibits higher inertial effects at elevated electrolyte velocities compared to the electrode with groove patterns under FTFF, possibly due to increased flow disturbances and non‐linear pressure dynamics.^[^ ^19^ ^]^

**Figure 4:**
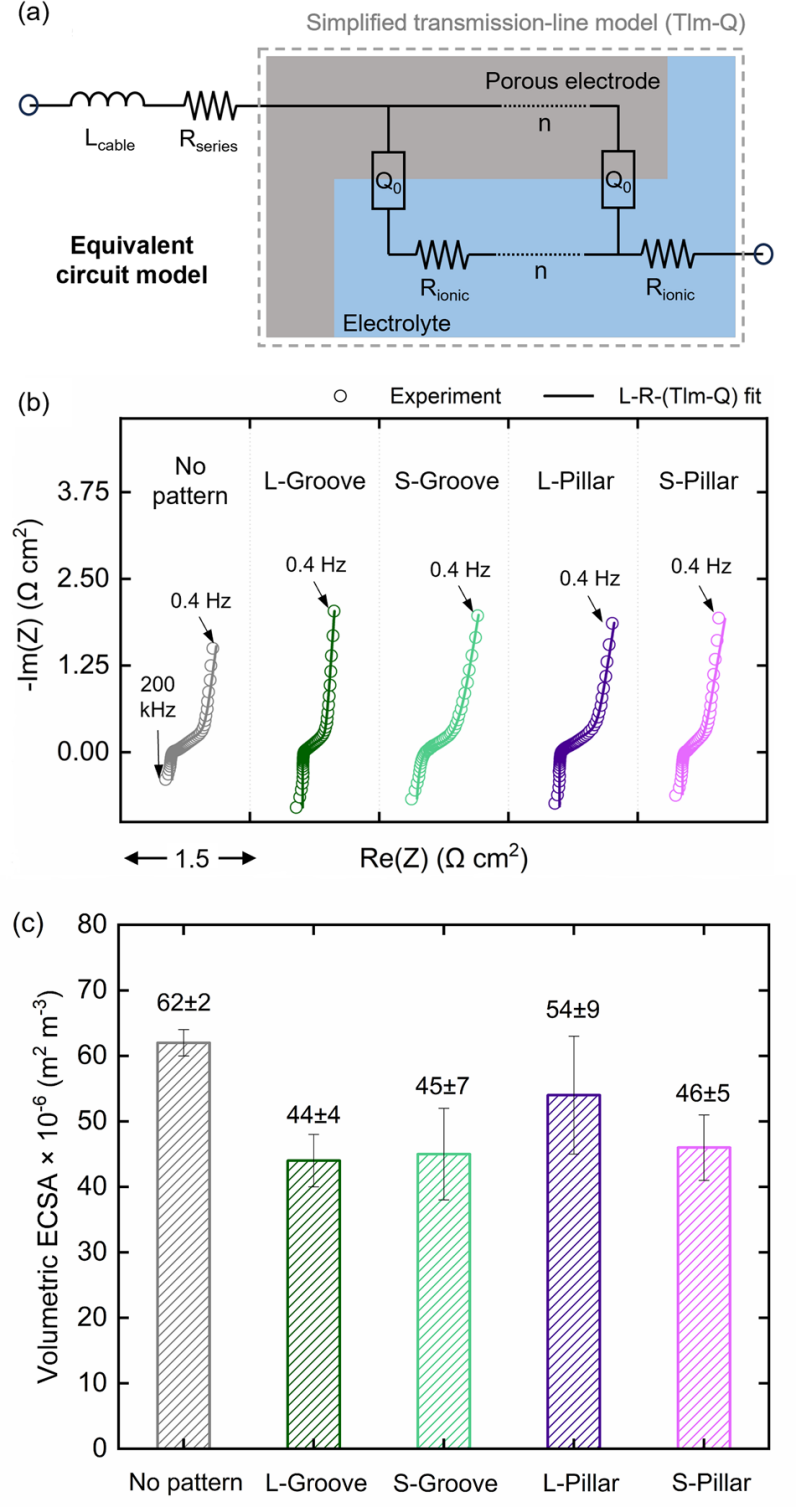
Estimation of the ECSA in the micro‐patterned NIPS electrodes. a) Equivalent circuit model used in EIS fitting. b) Nyquist plots obtained through EIS measurements (dots) and the corresponding fits (lines) at *v*_e_ ≈5 cm s^−1^ in a 2 m HCl solution. c) Volumetric ECSAs obtained from the fitted EIS data for each micro‐patterned electrode. The error bars correspond to a standard deviation (*n* = 2).

Despite the benefit of reduced pressure drop from adding groove or pillar patterns, removing electrode material in the patterned regions also raises concerns about decreased active surface area due to material removal in the patterned regions. To assess this trade‐off, we measure the ECSA of each sample using EIS and the equivalent circuit model depicted in **Figure** **4**a. The EIS fitting results are presented in Figure 4b, and the corresponding ECSA values are summarized in Figure 4c.

The results show that all sponge‐like NIPS electrodes exhibit high volumetric ECSA (e.g., 62 × 10^6^ m^2^ m^−3^ / 220 m^2^ g^−1^ for the no‐pattern electrode), attributed to their small pore sizes and narrow pore size distribution. To validate the accuracy of ECSA estimations by EIS, we perform cyclic voltammetry on the no‐pattern NIPS electrode (Figure S12, Supporting Information), obtaining a comparable value of 48 × 10^6^ m^2^ m^−3^ (170 m^2^ g^−1^). The difference between the two techniques arises from the assumptions in their models. In EIS, the electrode‐electrolyte interface is modeled using a CPE element, which accounts for non‐ideal capacitive behavior arising from surface roughness, porosity, and spatial heterogeneity. In contrast, CV‐based ECSA estimation assumes an ideal double‐layer capacitor and is more sensitive to the wetted, ion‐accessible surface area, leading to slight discrepancies in the extracted ECSA values.^[^ ^89^ ^]^ The micro‐patterned electrodes generally show lower ECSAs than the no‐pattern electrodes, reflecting the loss of electrode material due to micro‐patterning. Notably, the reduction in volumetric ECSA exceeds the ratio of electrode volume loss (see Table S3, Supporting Information), likely because of shrinkage and deformation of the micro‐patterns during carbonization. SEM images also reveal small holes at the borders of grooves and pillars (Figure 2a,b), indicating additional material loss. Overall, a properly designed micro‐pattern can substantially reduce flow resistance and thereby lower pumping requirements. However, excessive material removal can lead to significant ECSA loss, underscoring the need to balance hydraulic performance with electrochemical activity when designing micro‐patterned electrodes.

We observe that the ECSAs measured in this study are higher than those reported for the finger‐shaped NIPS electrodes (e.g., ≈1‐2 m^2^ m^−3^) in our previous work.^[^ ^66^, ^67^, ^68^ ^]^ Beyond the contribution from smaller pore sizes in the sponge‐like NIPS structure, we anticipate that oxygen infiltration during carbonization may create nanoscale surface defects,^[^ ^90^ ^]^ thereby enhancing the ECSA. Similar trends in ECSA enhancement are also reported when thermally treating commercial PAN‐based carbon electrodes. For example, the ECSA of SGL 29AA carbon paper increases from 1 m^2^ g^−1^ in its pristine state to ≈50 m^2^ g^−1^ after thermal treatment in air at 400 °C for 24 h.^[^ ^39^, ^40^ ^]^ To verify the role of oxygen, we employ XPS to estimate the oxygen content in all sponge‐like NIPS electrodes (Figure S13, Supporting Information). An atomic survey (Table S6, Supporting Information) reveals significantly higher oxygen content (O at% >11%) compared to the finger‐shaped NIPS electrodes from our previous study (O at% ≈ 4%).^[^ ^66^, ^67^, ^68^ ^]^ This elevated oxygen level likely explains the higher ECSA. Oxygen incorporation can benefit redox flow batteries by mitigating kinetic resistances,^[^ ^91^ ^]^ although excessive thermal treatment risks considerable mass loss and diminished cell performance.^[^ ^39^, ^40^ ^]^ Consequently, carefully controlling oxygen levels during carbonization is an effective strategy for optimizing both electrode nanoscale roughness and battery performance in future studies. In addition, the long‐term stability of oxygen content under practical battery cycling conditions remains a potential concern, as its degradation may alter surface properties and compromise overall electrode durability.^[^ ^92^, ^93^ ^]^

### 3.3 Electrochemical Performance of the Electrodes with Micro‐Grooves

To further evaluate the electrochemical performance of the micro‐patterned NIPS electrodes, we first characterize them in an iron symmetric cell, an effective platform for assessing electrode kinetics and mass transport while minimizing convoluting phenomena (e.g., crossover, side reactions).^[^ ^66^, ^67^, ^68^ ^]^ We investigate the influence of micro‐patterns on charge and mass transfer processes by subtracting the ohmic resistance (*R*_Ω_) from all EIS spectra and applying an *iR*_Ω_ correction to the polarization curves. The measured *R*_Ω_ ranges between 0.20 and 0.27 Ω for all NIPS electrodes. Variations in ohmic resistance among different electrode samples may arise from factors such as contact resistance, differences in electrode thickness, or variations in compression ratio. The subtracted ohmic resistances are summarized in Table S7 (Supporting Information), and the corresponding effects on the polarization curves are shown in Figure S14 (Supporting Information). Details of the EIS fitting parameters are provided in Table S8 (Supporting Information).

**Figure 5:**
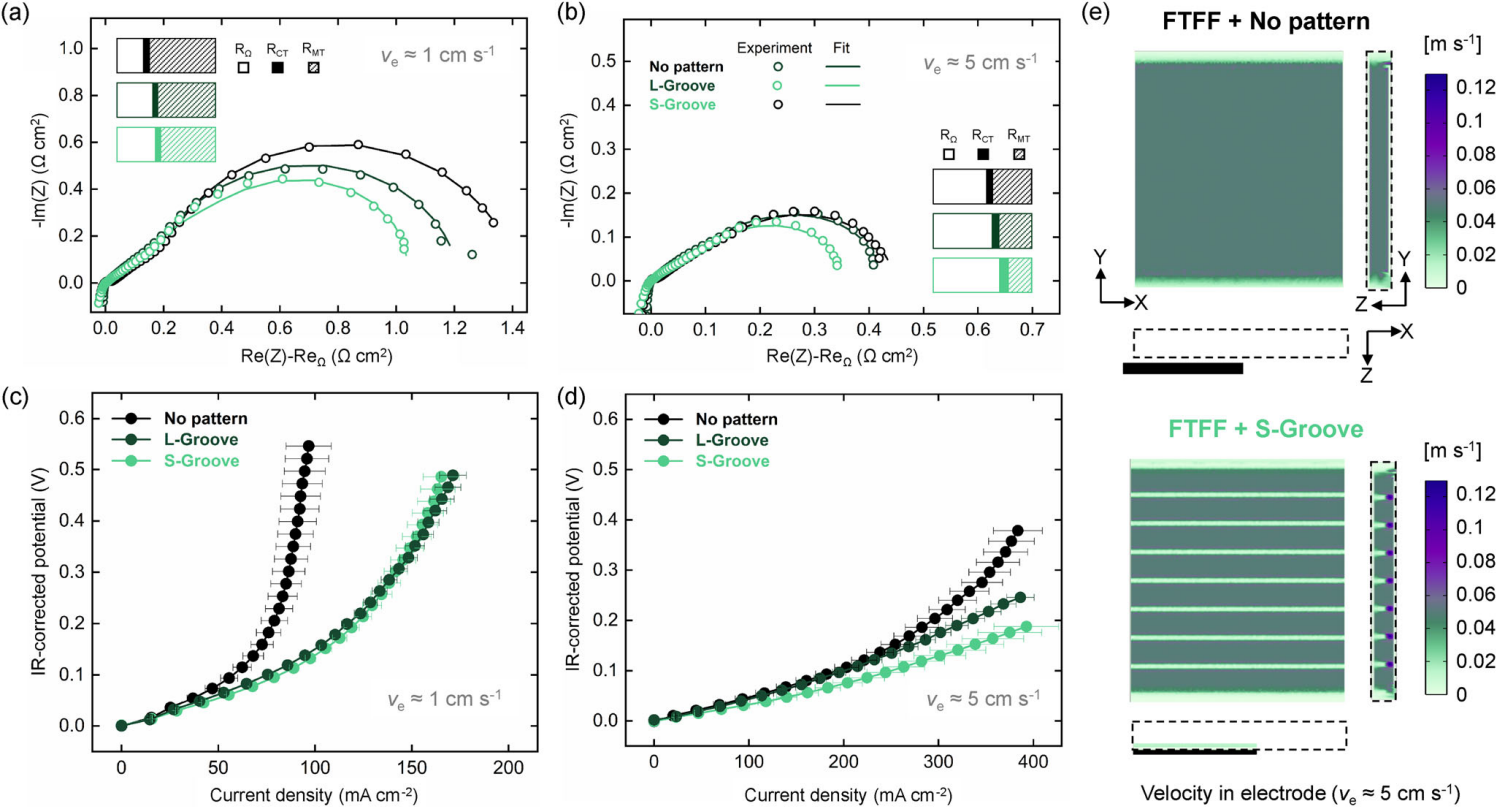
Electrochemical performance of the NIPS electrodes with micro‐grooves in a 0.5 M Fe^2+^/Fe^3+^ symmetric cell under FTFFs. a,b) Nyquist plots obtained through EIS measurements (dots) and the EIS fittings (lines) at *v*_e_ ≈1 and 5 cm s^−1^ after subtracting the ohmic resistances. c,d) IR_Ω_‐corrected polarization curves. The error bars correspond to a standard deviation (*n* = 2). e) Numerical results showing velocity distribution maps at *v*_e_ ≈5 cm s^−1^, derived from planar cross‐sections taken at half the electrode thickness (XY plane), along the electrode width (YZ plane), and along the electrode length (XZ plane).

The performance of the no‐pattern NIPS electrodes is initially assessed to establish a baseline for evaluation of the micro‐patterned designs. In **Figure** **5**, we show results at electrolyte velocities of 1 and 5 cm s^−1^, while the complete range of flow velocities is presented in Figure S15 (Supporting Information). Note that achieving an electrolyte velocity of 10 cm s^−1^ was not feasible for no‐pattern NIPS electrodes, which leads to severe battery leakage. However, introducing groove patterns significantly reduces the pressure drop (see Figure 3), enabling an electrolyte velocity of 10 cm s^−1^ without leakage.

As revealed by the EIS spectra (Figure 5a,b), the mass transfer resistance (*R*_MT_) contributes to a major portion of the total resistance, particularly for the no‐pattern sponge‐like NIPS electrodes. For instance, the mass transfer resistance is fitted as ≈1.4 Ω cm^2^ at *v*_e_ ≈1 cm s^−1^ for the no‐pattern electrodes, accounting for ≈67% of the total resistance. The *R*_MT_ remains substantial for the no pattern electrodes at an elevated *v*_e_ of 5 cm s^−1^. In contrast, the kinetic resistance (*R*_CT_) at 1 and 5 cm s^−1^ is comparatively low (e.g., ≈6% of the total resistance), likely due to the high electrochemical surface area and surface functionalities of the sponge‐like NIPS. Similar trends are also observed in the polarization curves: while the current response appears nearly linear at low overpotentials, the achievable current density (e.g., ≈100 mA cm^−2^ at 1 cm s^−1^) is constrained by rapid species depletion. Consequently, mitigating *R*_MT_ is critical for enhancing the electrochemical performance of no‐pattern sponge‐like NIPS electrodes.

For the micro‐patterned electrodes, EIS spectra indicate that incorporating groove designs slightly reduces the overall cell resistance, primarily through lowering the mass transfer resistance. At *v*_e_ ≈1 cm s^−1^, the combined kinetic and mass transfer resistances for the S‐Groove design (≈1.0 Ω cm^2^) are lower than those for the L‐Groove design (≈1.2 Ω cm^2^), and both are below those of the no pattern electrode (≈1.4 Ω cm^2^). This improvement is predominantly attributed to a decrease in mass transfer resistance. For instance, at *v*_e_ ≈1 cm s^−1^, the mass transfer resistance of the S‐Groove electrode is ≈25% lower than that of the no pattern electrode, while the kinetic resistance remains unchanged. Moreover, the polarization curves (Figure 5c,d) show that the early‐reaching mass transport limitations observed in the no‐pattern NIPS electrodes are largely mitigated by the groove micro‐patterns, facilitating higher current densities. Among the three designs, the patterned electrode (S‐Groove) achieves the lowest overall overpotentials at the same flow velocities, reflecting a more effective reduction in mass transfer resistance and enhanced electrochemical performance. At *v*_e_ ≈5 cm s^−1^, the mass transfer resistance constitutes only ≈23% of the total resistance, with the majority arising from ohmic losses.

To provide deeper insight into the experimental observations and to visualize the electrolyte distribution within both porous electrode and flow field, the pressure streamlines resulting from the simulations (Figure S16, Supporting Information) and 2D electrolyte velocity distribution contours within the electrode structures (Figure 5e) are analyzed in the 3D porous domain using the continuum 3D half‐cell model at *v*_e_ ≈5 cm s^−1^. The 2D contours are taken from a cross‐section at half the electrode thickness. This model is applied to the S‐Groove design coupled with FTFF, which has demonstrated better electrochemical performance. For comparative analysis, the results of the no‐pattern electrodes are also presented.

The numerical results reveal that for the patterned NIPS electrodes (S‐Groove) under FTFF configuration, the pressure drop is largely decreased compared to the non‐patterned electrodes, which is in good agreement with the experimental findings. The presence of micro‐grooved channels within the electrode leads to a decreased volume of porous electrode that the electrolyte needs to flow through, reducing the pressure drop. Additionally, enhanced electrolyte mixing along the electrolyte flow pathway is observed from the longitudinal view (YZ) as shown in Figure S16 (Supporting Information). The improved electrolyte mixing, as revealed in the fluid dynamic analysis, may result in a more uniform distribution of species within the patterned electrodes (S‐Groove). Additionally, the velocity distribution map clearly shows that the electrolyte preferentially flows within the groove‐patterned regions (see YZ XZ plane views in Figure 5e). The average electrolyte velocity of the grooved regions in the patterned electrode (S‐Groove) is simulated as 7.5 cm s^−1^. Effective species conversion in these areas is driven by the enhanced electrolyte mixing can help to avoid stagnant regions. This improvement helps to reduce concentration overpotentials and boost the electrochemical performance.

We additionally evaluate how changing the alignment between the grooved micro‐channels in the electrode and the flow direction imposed by the FTFF affects performance (Figure S17, Supporting Information). Although the aligned groove orientation lowers the pressure drop, it substantially increases mass transfer resistance. We speculate that the electrolyte tends to flow along the surface groove channels of the sponge‐like NIPS electrode rather than penetrating deeply into its porous structure, thereby limiting effective mass transfer.

### 3.4 Electrochemical Performance of the Electrodes with Micro‐Pillars

**Figure 6:**
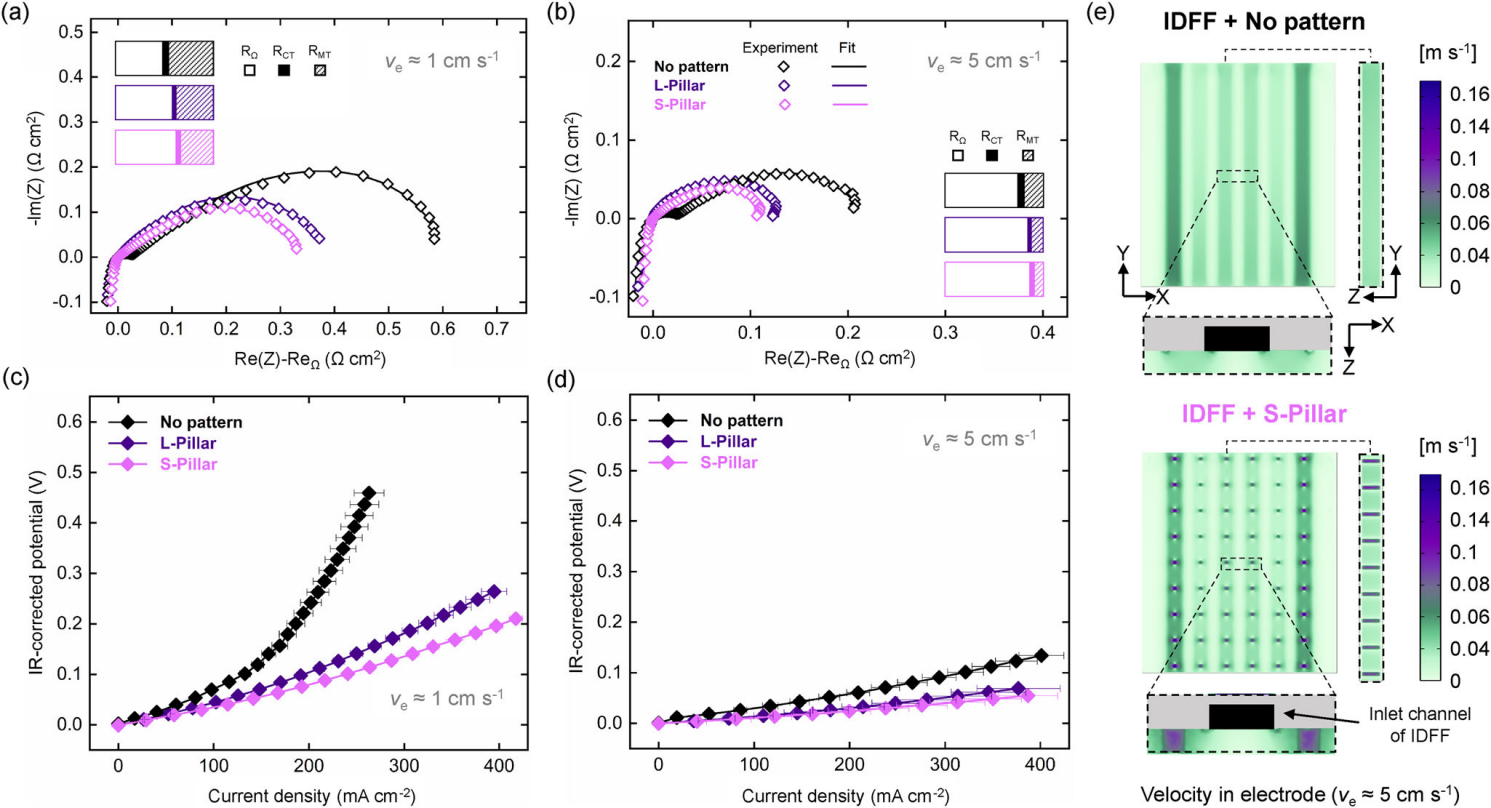
Electrochemical performance of the NIPS electrodes with micro‐pillars in a 0.5 m Fe^2+^/Fe^3+^ symmetric cell under IDFFs. a,b) Nyquist plots obtained through EIS measurements (dots) and the EIS fittings (lines) at *v*_e_ ≈1 and 5 cm s^−1^ after subtracting the ohmic resistances. c,d) IR_Ω_‐corrected polarization curves. The error bars correspond to a standard deviation (*n* = 2). e) Numerical results showing velocity distribution maps at *v*_e_ ≈5 cm s^−1^, derived from planar cross‐sections taken at half the electrode thickness (XY plane) and half the electrode length (XZ plane), with the YZ plane taken along a row of micro‐pillars.

The electrochemical performance of the sponge‐like NIPS electrodes with micro‐pillars in an IDFF configuration at *v*_e_ ≈1 and ≈5 cm s^−1^ is shown in **Figure** **6**. The results at other flow velocities are shown in Figure S18 (Supporting Information). The performance of the no‐pattern NIPS electrodes with IDFF is provided for comparison. Details of the EIS fitting parameters are provided in Table S9 (Supporting Information).

It is observed that high electrolyte velocities up to 10 cm s^−1^ can be achieved for no pattern sponge‐like NIPS electrodes without battery leakage, in contrast to the combination with FTFFs, which results in high pressure drop and leakages. As previously reported,^[^ ^19^ ^]^ when electrodes featuring a homogeneous microstructure and small pore sizes are combined with IDFFs, the electrolyte is supplied at intermediate points in the electrode, promoting the replenishment of species, reducing the residence time, and reducing pressure drop. As such, in the case of no pattern NIPS electrodes, we similarly observed a reduction in the combined kinetic and mass transfer resistances (e.g., ≈0.6 Ω cm^2^) under IDFF, compared to the FTFF configuration, which shows higher total resistance (e.g., ≈1.4 Ω cm^2^) at the same electrolyte velocity. The enhancement in performance is attributed to the reduction in mass transfer resistance facilitated by the forced convection between channels in the IDFF configuration.

Although lower total resistance is observed under IDFF configuration, incorporating micro‐pillars into the sponge‐like NIPS electrodes can further substantially reduce the mass transfer resistance, as shown in Figure 6a,b. The results show that the S‐Pillar design features lower electrochemical resistance than the L‐Pillar design. For instance, at the electrolyte flow velocity of 1 cm s^−1^, the mass transfer resistance decreases by ≈45% when comparing the no‐pattern electrodes to the patterned electrodes (S‐Pillar). The patterned electrodes (S‐Pillar) demonstrate high electrochemical performance with the total kinetic and mass transfer resistances of ≈0.3 Ω cm^2^ at 1 cm s^−1^. This resistance is further reduced to below 0.1 Ω cm^2^ at a linear electrolyte velocity of 10 cm s^−1^ (see Figure S18, Supporting Information). Furthermore, the polarization curves (Figure 6c,d) indicate that the regions of mass transfer limitation are not pronounced for the NIPS electrodes featuring micro‐pillars, even in areas of high current density and at low electrolyte flow velocities.

To better understand the reasons behind the promising electrochemical improvement of the pillar‐patterned electrodes, we analyze the pressure streamlines and flow distribution maps obtained from the cell continuum model at *v*_e_ ≈5 cm s^−1^. These visualizations help illustrate how the micro‐pillars influence electrolyte flow and pressure distribution, contributing to the observed performance enhancement. For the patterned NIPS electrodes (S‐Pillar) with IDFF configuration, the pressure drop streamlines do not show a significant reduction in pressure drop when compared to the no‐pattern electrodes (Figure S16, Supporting Information), which is in accordance with our experimental findings (see Figure 3b). However, the streamlines indicate improved electrolyte mixing, which is particularly evident in the micro‐pillar regions between the inlet and outlet channels in IDFF. The transversal view (XZ) for a zoomed outlet channel and two ribs evidences a more homogeneous distribution of the flow streamlines within the under‐rib regions of the patterned electrodes (S‐Pillar). In addition, a higher local electrolyte velocity is observed at the pillar regions in patterned electrodes (S‐Pillar) as shown from the XZ view in Figure 6e, compared to the non‐patterned electrodes. This indicates that the design of the patterned electrodes (S‐Pillar) significantly enhances under‐rib convection along the electrolyte flow pathways, which can lead to more efficient conversion of active species,^[^ ^94^, ^95^ ^]^ which helps explain the enhanced electrochemical performance as shown in Figure 6a–d. Our findings show that appropriately designed micro‐patterns within the electrode structure, along with a synergistic optimization between flow fields and electrode channels or patterns, can significantly enhance flow battery performance.

### 3.5 Balancing the Electrical Power and Pumping Loss

To comprehensively evaluate the performance enhancement and trade‐offs of micro‐patterned NIPS electrodes compared to no‐pattern electrodes, we analyze both the electrical power output and the corresponding pumping power required for different electrode configurations. Specifically, we select the micro‐patterned electrodes with higher performance in each configuration, S‐Groove under FTFF and S‐Pillar under IDFF, and compare them with the no‐pattern electrodes under the same flow field conditions. To estimate the electrical power for an all‐iron RFB application, we adopt an equilibrium potential of 1.21 V.^[^ ^96^, ^97^, ^98^ ^]^ For simplification, we assume that the overpotential is symmetric and corresponds to that of the Fe^2+^/Fe^3+^ half‐cell, as measured in the iron symmetric cell shown in Figures 5 and 6. We acknowledge that this is a simplification, but it serves the purpose of this theoretical exercise. The electrical power is calculated from the current output at 0.1 V overpotential extracted from the polarization curves without iR‐correction at the electrolyte velocity of 1 cm s^−1^. The equation is expressed as follows:

(5) $$P_{electrical}=\left(1.21-\eta \right)\times I$$

where

*P_electrical_* denotes electrical power;

*η* denotes overpotential;

*I* denote current.

The corresponding pumping power is estimated based on the pressure drop measurements shown in Figure 3 at the electrolyte velocity of 1 cm s^−1^. We assume the pump efficiency of 0.8.^[^ ^99^ ^]^ The equation is expressed as follows:

(6) $$P_{pump}=\frac{Q\times \Delta P}{\eta_{pump}}$$

where

*P_pump_* denotes pumping power;

*η_pump_* denotes pump efficiency.

**Figure 7:**
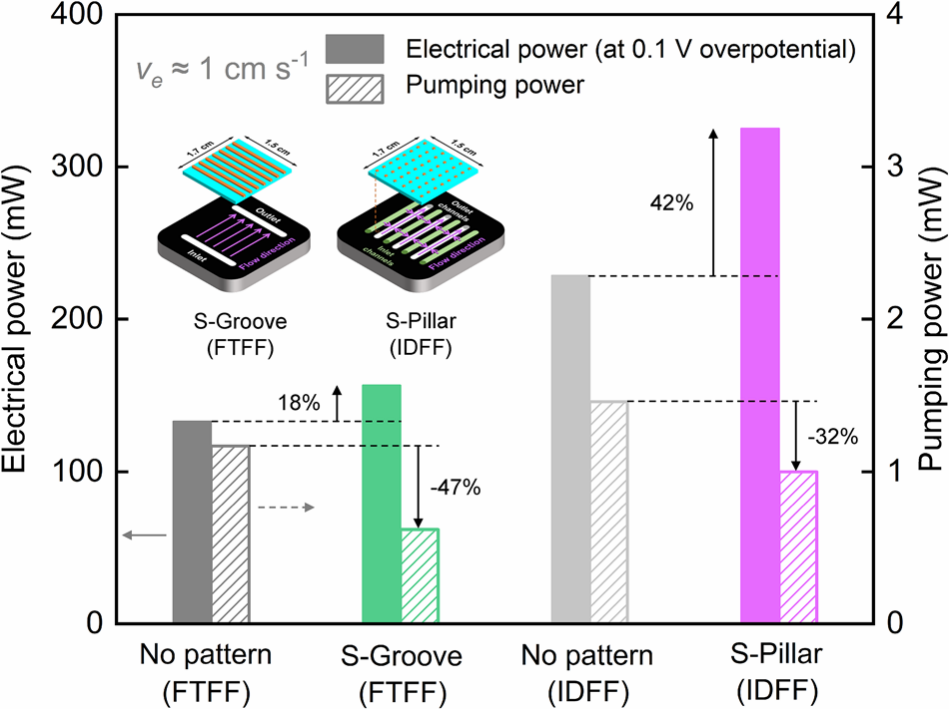
Electrical power calculated from the polarization curves at 0.1 V overpotential in a 0.5 M Fe^2+^/Fe^3+^ symmetric cell at *v*_e_ ≈1 cm s^−1^, alongside the corresponding pumping power required for the four different electrode configurations.

The performance gains of the patterned electrodes (S‐Groove and S‐Pillar) are summarized in **Figure** **7**. The micro‐patterned NIPS electrodes improve electrical power output and reduce pumping losses compared to the no‐pattern electrodes under both FTFF and IDFF configurations. Specifically, under FTFF, the patterned electrode (S‐Groove) increases electrical power by 18% and reduces pumping power loss by 47% at *v*_e_ ≈1 cm s^−1^. The improvement is more pronounced for the patterned electrode (S‐Pillar) under IDFF, where the electrical power is increased by 42% and the required pumping power is reduced by 32% compared to the no‐pattern electrode. These enhancements are attributed to improved mass transport and fluid dynamics enabled by the integration of micro‐patterns within the electrode structure.

### 3.6 Vanadium Full‐Cell Tests

**Figure 8:**
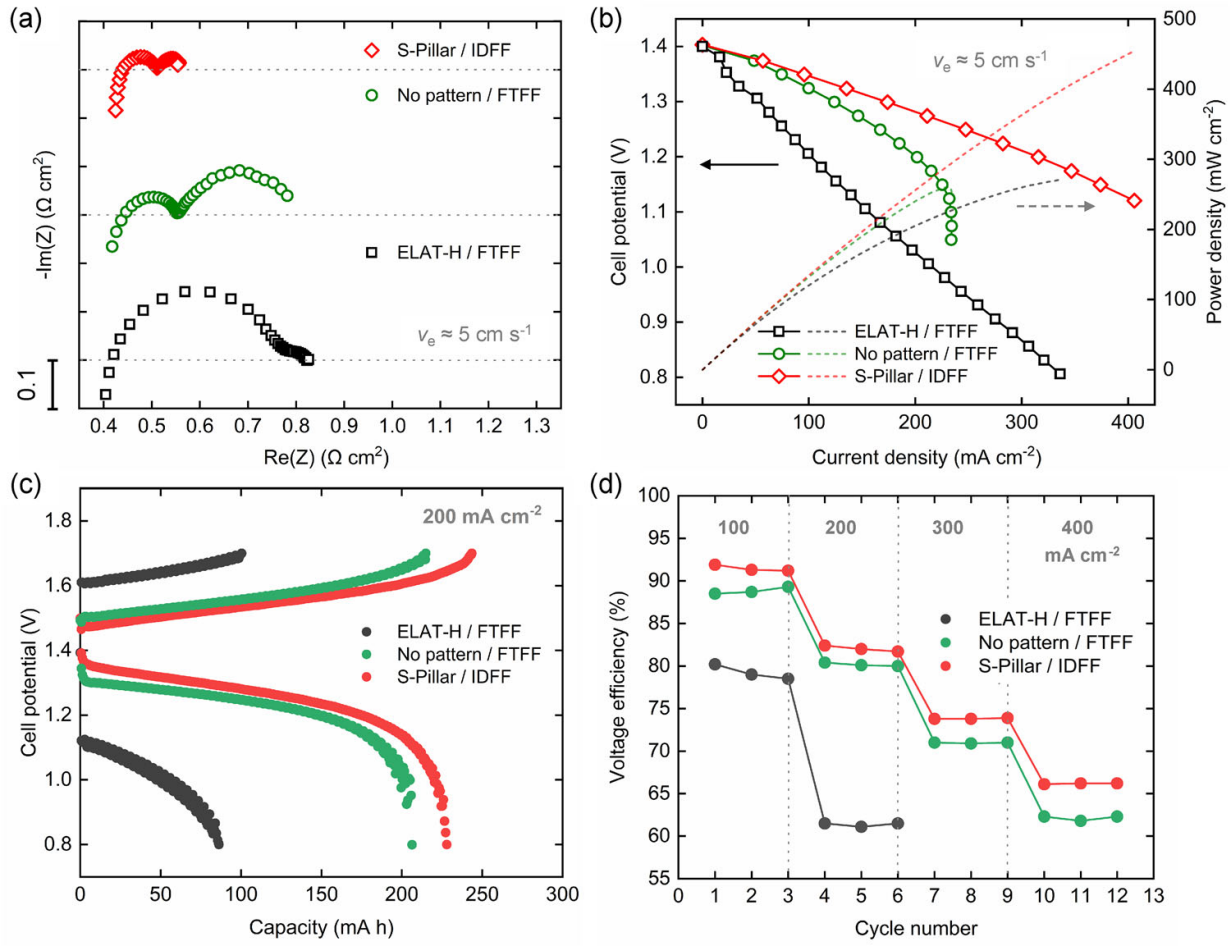
Electrochemical performance comparison of the S‐Pillar NIPS electrodes with a thermally treated ELAT‐H carbon cloth electrode in a 1.6 m vanadium full‐cell. a) EIS spectra and (b) discharge polarization curve at *v*_e_ ≈5 cm s^−1^ at 50% SoC. c) Charge and discharge profiles at 200 mA cm^−2^ and d) voltage efficiency during battery cycling at varying applied current densities.

To benchmark the performance of our electrodes against the current state‐of‐the‐art, we conduct a comparative evaluation using all‐vanadium full‐cell testing. For this, we select the best‐performing micro‐patterned design in the present study, the patterned electrodes (S‐Pillar), for a comparative evaluation. The electrochemical performance is compared to a commercial carbon cloth electrode with a similar electrode thickness (e.g., ELAT‐H) at an electrolyte velocity of 5 cm s^−1^ in a vanadium full cell setup. For fair comparison, the ELAT‐H is thermally treated at 450 °C for 12 h to ensure hydrophilicity.^[^ ^39^, ^40^ ^]^ The performance of the no pattern NIPS electrodes under FTFF is also presented for comparison, and the results are summarized in **Figure** **8**. A broader comparison with commercial carbon‐fiber electrodes is provided in Figure S19 (Supporting Information).

From the EIS spectra presented in Figure 8a, it is observed that the NIPS electrodes exhibit ohmic resistances comparable to those of the commercial ELAT‐H carbon cloth. The ELAT‐H shows the highest overall resistance, where substantial losses are predominantly due to the kinetic resistance. This is primarily due to its low ECSA (≈1 m^2^ g^−1^),^[^ ^88^ ^]^ which remains lower than NIPS electrodes even after thermal treatment. Conversely, the carbon cloth benefits from its woven structure and bimodal pore size distribution, which promotes greater mass transport of species. These characteristics have established carbon cloth as a favorable option for RFB applications in recent years.^[^ ^33^ ^]^ However, there are still challenges that need to be addressed to fully leverage its potential.

In comparison, the no‐pattern sponge‐like NIPS electrodes display a similar overall resistance of ≈0.8 Ω cm^2^. However, their predominant potential loss stems from mass transfer resistance, due to the small pore sizes and narrow pore size distribution. The impact of mass transfer limitations is further evident in the discharge polarization curves shown in Figure 8b. The no pattern sponge‐like NIPS electrodes exhibit lower overpotentials in low current density regions compared to the ELAT‐H carbon cloth. However, they encounter mass transfer limitation regions at higher current densities, where the electrochemical performance quickly deteriorates.

The patterned electrodes (S‐Pillar) coupled with IDFF configuration emerge as the best‐performing combination in the present study. They exhibit optimal electrochemical performance, characterized by both minimized kinetic resistance and low mass transfer resistance. The overall resistance is <0.6 Ω cm^2^, including an ohmic resistance of 0.43 Ω cm^2^, which primarily arises from membrane resistance and contact resistance in RFBs. The overpotential remains below 0.05 V at a current density of 100 mA cm^−2^ during discharge polarization measurements, and a power density of 450 mW cm^−2^ is achieved at a current density of 400 mA cm^−2^.

From the cycling results shown in Figure 8c,d, the S‐Pillar electrode shows the best performance among the three electrode configurations. Specifically, it delivers more than twice the charge and discharge capacity compared to the thermally treated carbon cloth electrode at 200 mA cm^−2^. In terms of voltage efficiency, the micro‐patterned electrode (S‐Pillar) achieves a voltage efficiency of 92% at 100 mA cm^−2^, and 66% at 400 mA cm^−2^. These full‐cell cycling results confirm that the performance enhancements observed in EIS and polarization measurements translate into improved battery‐level performance. Longer‐term battery cycling to evaluate lifetime and commercial‐scale demonstration will be considered in future work.

To summarize, the incorporation of micro‐patterns within the electrode architecture shows significant promise for enhancing battery performance. This strategy contributes to both reduced pressure drop and improved electrochemical performance based on the NIPS electrodes, indicating a promising avenue for the enhancement and optimization of high‐performance RFB systems.

## 4 Conclusion

In the present study, the non‐solvent induced phase separation technique was employed to synthesize sponge‐like NIPS electrodes under slow phase inversion conditions. During this process, micro‐patterns, including groove and pillar designs, were integrated into the electrode architecture. The resulting sponge‐like NIPS electrodes feature small pore sizes and a narrow pore size distribution, contributing to a high volumetric specific surface area but low permeability. The incorporation of micro‐patterns into the electrode structure significantly enhances electrolyte penetration, leading to a marked reduction in pressure drop and an improvement in mass transfer. Numerical results indicate that both micro‐grooves and micro‐pillars are effective in enhancing electrolyte mixing, thereby improving local mass transfer within the electrodes compared to the no‐pattern electrodes. Notably, patterned electrodes (S‐Pillar), when coupled with the IDFF, exhibit outstanding electrochemical performance by significantly reducing both kinetic and mass transfer resistances. Specifically, in an iron symmetric redox flow cell, the total resistance of both kinetic and mass transfer is <0.1 Ω cm^2^ at an electrolyte velocity of 10 cm s^−1^. When compared to state‐of‐the‐art electrodes, such as a thermally activated ELAT‐H carbon cloth, in an all‐vanadium RFB, the total resistance of kinetic and mass transfer is reduced by ≈65%. We demonstrate that micro‐patterned electrodes fabricated via NIPS can improve electrolyte transport, reduce pressure drop, and enhance electrochemical performance compared to conventional carbon‐fiber‐based electrodes. This approach offers a scalable and cost‐efficient pathway to integrate complex 3D flow patterns into electrode architectures, contributing to the development of more efficient RFBs for large‐scale energy storage applications.

## Conflict of Interest

The authors declare no conflict of interest. B.L., R.R.‐J., J.H., and A.F.‐C. have submitted a patent related to this work.

## Author Contributions

B.L. contributed to the conceptualization, methodology, formal analysis, investigation, data curation, writing‐original draft, writing‐review and editing, and visualization. R.R.‐J. contributed to methodology, formal analysis, investigation, data curation, and writing‐review and editing. V.M.‐P. contributed to software, validation, formal analysis, visualization, and writing‐review and editing. S.B. contributed to data curation and writing‐review. J.H. contributed to methodology, writing‐original draft, writing‐review and editing, and supervision. Finally, A.F.‐C. contributed to the conceptualization, methodology, funding, resources, writing‐original draft, writing‐review and editing, project administration, and supervision.

## Supporting information



Supporting Information

## Data Availability

The data that support the findings of this study are available from the corresponding author upon reasonable request.
