# Supplementary material for: Integrating Flow Field Geometries within Porous Electrode Architectures for Enhanced Flow Battery Performance
Source: Small. 2025 Oct 24;22(1):e11327. doi: 10.1002/smll.202511327 (PMC12757986; doi:10.1002/smll.202511327)
Supplement: Supplementary file 1 — Supporting Information [file SMLL-22-e11327-s001.pdf]

NANO · MICRO
small

Supporting Information

for *Small*, DOI 10.1002/smll.202511327

Integrating Flow Field Geometries within Porous Electrode Architectures for Enhanced Flow Battery Performance

*Baichen Liu, Rémy Richard Jacquemond, Vanesa Muñoz-Perales, Simona Buzzi, Johan Hjelm and Antoni Forner-Cuenca**

Supplementary Information

Integrating Flow Field Geometries within Porous Electrode Architectures for Enhanced Flow Battery Performance

Baichen Liu[a,b], *Rémy Richard Jacquemond*[a], *Vanesa Muñoz-Perales*[c], *Simona Buzzi*[a], *Johan Hjelm*[b], *Antoni Forner-Cuenca*[a*]

[a] Department of Chemical Engineering and Chemistry, Eindhoven University of Technology, 5600 MB Eindhoven, The Netherlands

[b] Department of Energy Conversion and Storage, Technical University of Denmark, DK-2800 Kgs. Lyngby, Denmark

[c] Electrochemical Processes Unit, IMDEA Energy, Avda. Ramón de La Sagra 3, 28935 Móstoles, Spain

[*] Corresponding author: a.forner.cuenca@tue.nl

**Author**	**ORCID number**	**Email address**
Baichen Liu	0000-0003-2250-4042	b.liu@tue.nl
Rémy Richard Jacquemond	0000-0002-4788-2915	r.r.jacquemond@tue.nl
Vanesa Muñoz-Perales	0000-0002-9691-918X	vamunoz@mit.edu
Simona Buzzi	0009-0007-9521-7921	simona.buzzi@kuleuven.be
Johan Hjelm	0000-0003-0072-5784	johh@dtu.dk
Antoni Forner-Cuenca	0000-0002-7681-0435	a.forner.cuenca@tue.nl

Table of Contents

1. Design of the micro-patterned molds 3

2. Microstructure of sponge-like and finger-shaped NIPS electrodes 4

3. Flow cell configuration for pressure drop and electrochemical measurements 5

4. Equivalent circuit model in EIS fitting 6

5. Establishment of cell continuum model 7

6. Model validation of pressure drop 9

7. Electrode shrinkage effects after carbonization 10

8. Volume loss ratios for micro-patterned electrodes 11

9. Permeability and non-linear Forchheimer coefficient of the NIPS electrodes 12

10. Water contact angle measurements 13

11. Mechanical compression tests 14

12. Pressure drop measurements of the empty cell 15

13. Cyclic voltammetry measurements 16

14. X-ray photoelectron spectroscopy (XPS) of electrode surface 17

15. Ohmic resistances of NIPS electrodes extracted from EIS measurements 18

16. Electrochemical performance of NIPS electrodes with micro-grooves 19

17. Pressure drop streamlines from cell continuum model 20

18. Effects of the flow direction in the groove-patterned NIPS electrodes 21

19. Electrochemical performance of NIPS electrodes with micro-pillars 22

20. EIS fitting parameters 23

21. Comparison with commercial carbon-fiber electrodes 24

22. References 25

1. Design of the micro-patterned molds

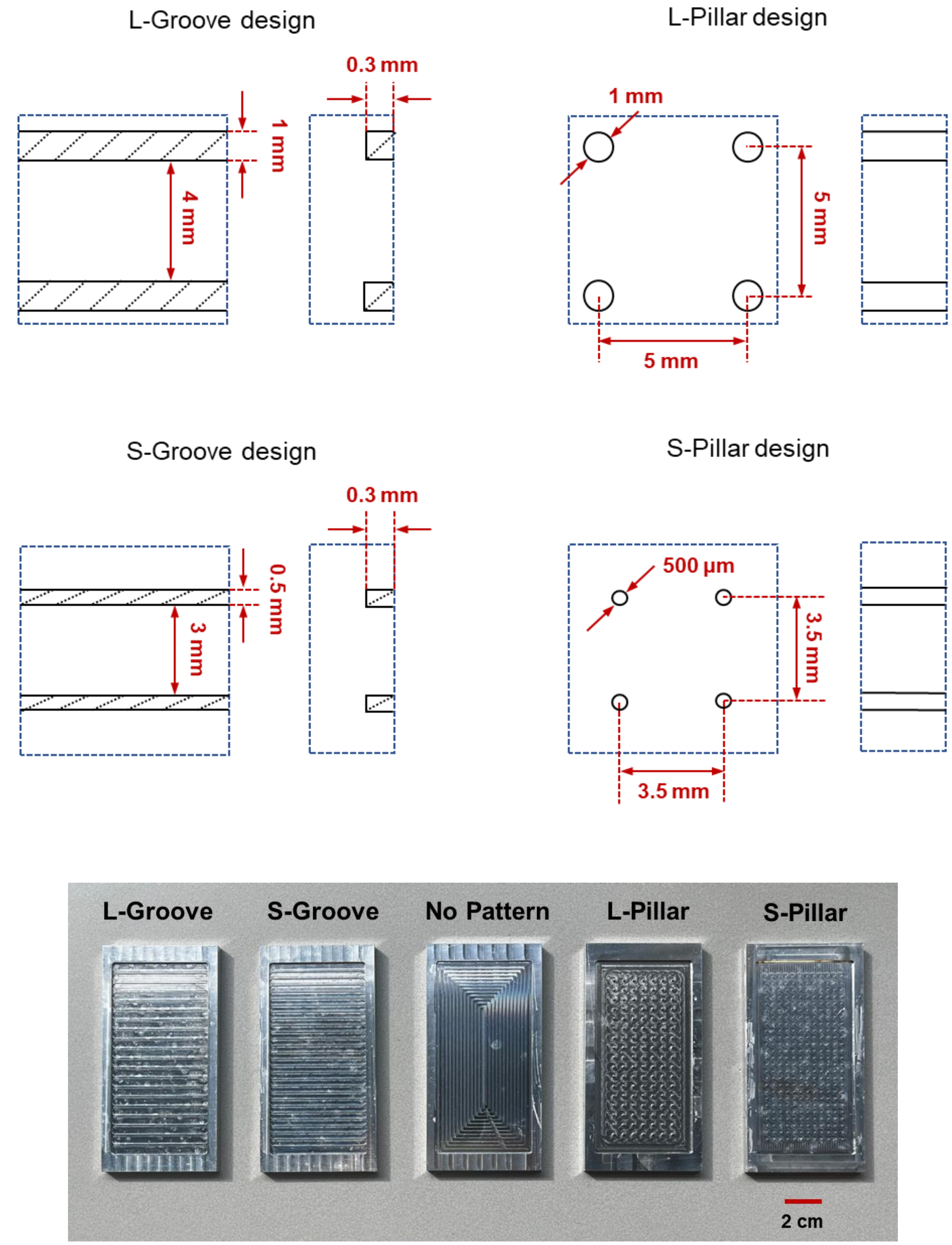

Figure S1. Schemes of the geometric sizes of the groove and pillar designs in micro-patterned molds, along with optical photographs of the aluminum molds.

2. Microstructure of sponge-like and finger-shaped NIPS electrodes

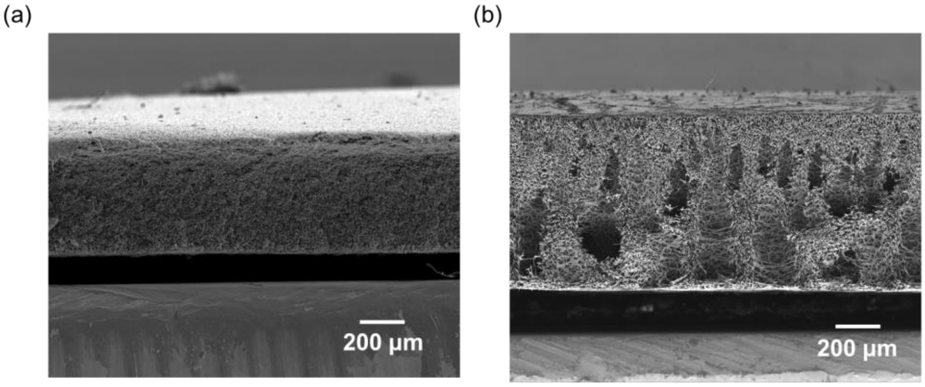

Figure S2. SEM images of the (a) no pattern sponge-like and (b) finger-shaped NIPS electrodes.

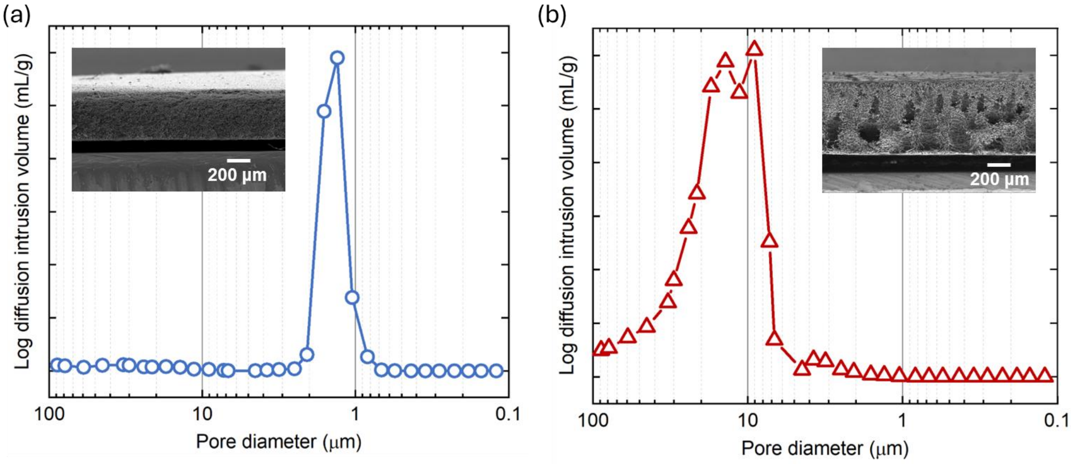

Figure S3. Pore size distribution of the no pattern (a) sponge-like and (b) finger-shaped NIPS electrodes measured from mercury intrusion porosimetry.

The measured porosities of the different electrode designs are 73.3% (no pattern), 72.9% (L-Groove), 76.1% (S-Groove), 74.9% (L-Pillar), and 72.2% (S-Pillar). These values indicate that the overall porosity remains comparable across all micro-patterned and no pattern NIPS electrodes, confirming that the introduction of micro-patterns does not significantly alter the internal sponge-like pore structure.

3. Flow cell configuration for pressure drop and electrochemical measurements

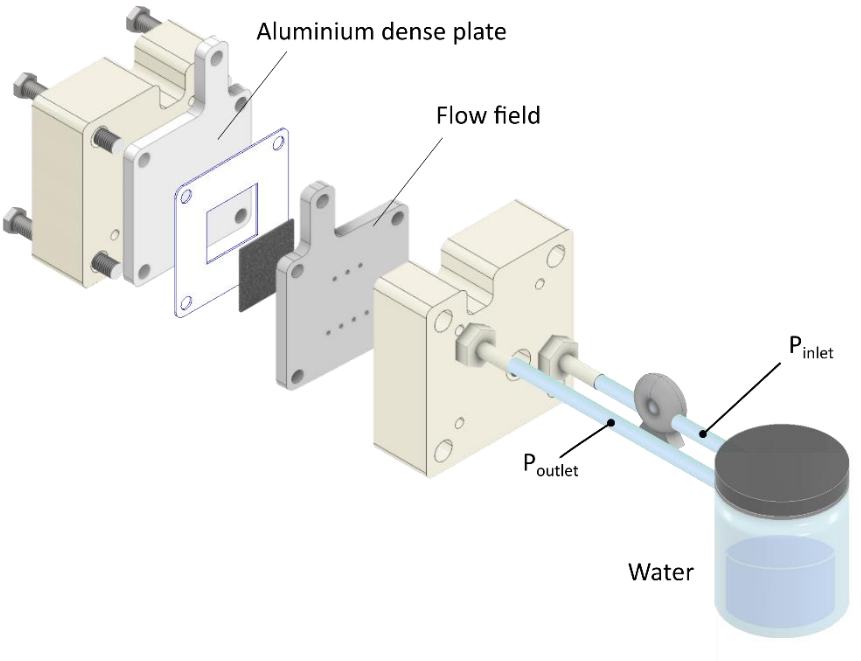

Figure S4. Cell configuration for pressure drop experiments [1].

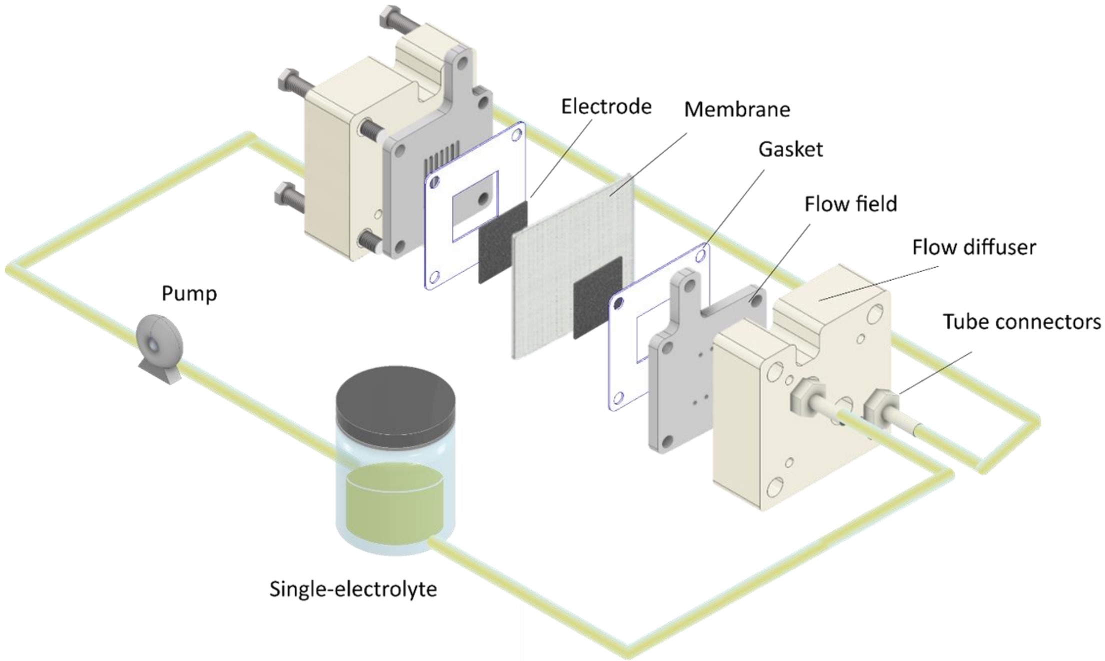

Figure S5. Single-electrolyte cell configuration for electrochemical experiments [1].

4. Equivalent circuit model in EIS fitting

EIS data is interpreted using an equivalent circuit model in the absence or presence of faradaic reactions, i.e. under blocking or non-blocking conditions. The model consists of an inductor to account for cable inductance, a resistor to represent membrane resistance, and a simplified transmission-line model (Tlm-Q) to describe the porous electrode. Under blocking conditions, the surface impedance of the interface between pores and electrolytes can be modelled with a CPE (Q_0) element, as shown in **Figure S6a**. Under non-blocking conditions, the Tlm-Q model incorporates a series of distributed elements to represent charge transfer kinetics at the pore surfaces and finite-length Warburg elements to capture diffusion processes within the pore network saturated with electrolyte, as shown in **Figure S6b**.

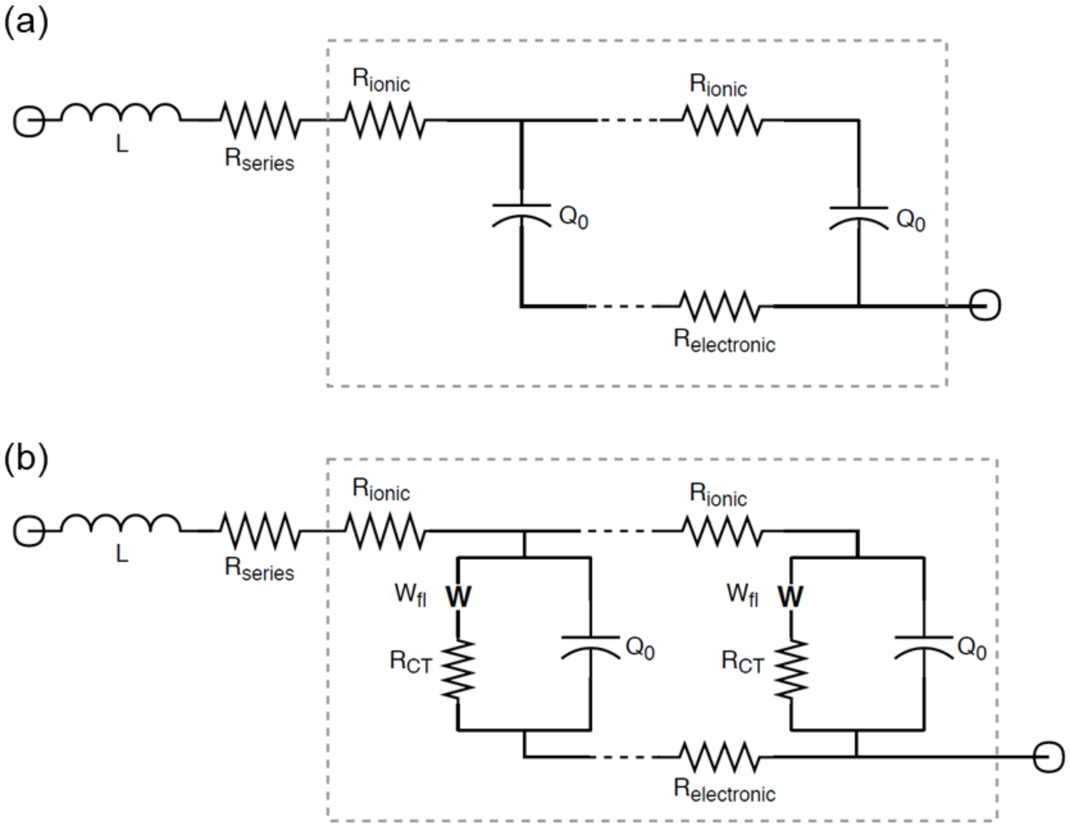

Figure S6. Schematic of the equivalent circuit used in the EIS fittings under (a) blocking and (b) non-blocking conditions in the present study. The circuit elements include inductor (L), resistor (R_{series}), and a transmission-line model. The electronic resistance ($R_{electronic}$) is assumed to be much lower than ionic resistance (R_{ionic}) and can be neglected in the simplified transmission-line model. Q_0 denotes surface capacitance of the interface between the electrolyte and the solid electrode.

5. Establishment of cell continuum model

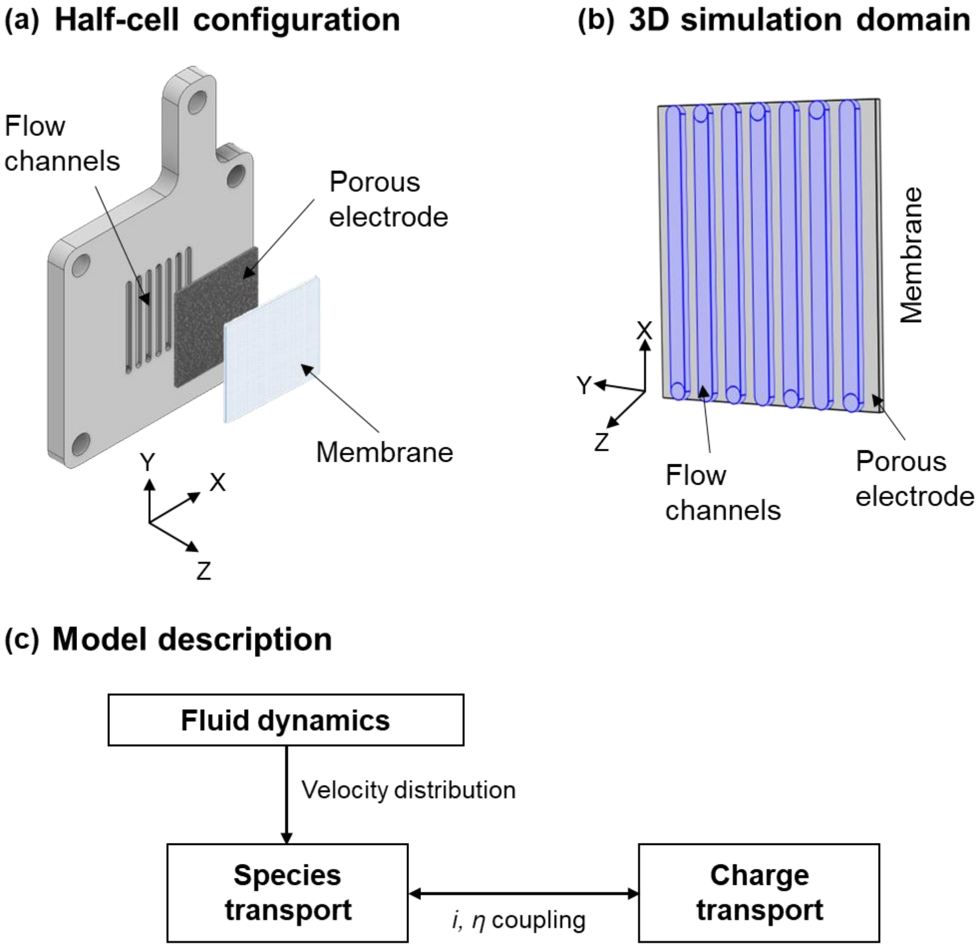

Figure S7. (a) Flow cell components used in simulation under a half-cell continuum model [2], with an interdigitated flow field as an example. (b) Geometrical domain for the finite elements simulation in COMSOL Multiphysics, with an interdigitated flow field and a no pattern NIPS electrode as an example. (c) Schematic diagram of the continuum 3D half-cell model. The fluid dynamics are first unidirectionally fed into the species and charge transport, and subsequently bidirectionally coupled.

Table S1 Numerical values adopted as input parameters in the numerical model

Symbol	Quantity	Value
δ	Electrode thickness [m]	4.2×10^{-4}
$ECSA$	Electrochemical surface area [m^2 m^{-3}]	6.2×10^{7}
k_L	Electrolyte conductivity [S m^{-1}]	30.84 [3]
σ_e	Electrode conductivity [S m^{-1}]	102.5 (measured)
ε	Porosity [-]	0.733 (measured)
κ	Permeability [m^2]	1.0×10^{-11} m
k^0	Reaction rate constant [cm s^{-1}]	1.84×10^{-5} [4]
α_a	Anodic transfer coefficient [-]	0.5 [5]
α_c	Cathodic transfer coefficient [-]	0.5 [5]
ρ	Electrolyte density [kg m^{-3}]	1015 [6]
μ	Electrolyte viscosity [Pa s]	1.143×10^{-3} [7]
$D_{Fe^{2+}}$	Diffusion coefficient of Fe^{2+} [m^2 s^{-1}]	5.7×10^{-10} [8]
$D_{Fe^{3+}}$	Diffusion coefficient of Fe^{3+} [m^2 s^{-1}]	4.8×10^{-10} [8]
$C_{0,Fe^{2+}}$	Initial concentration of Fe^{2+} [mol m^{-3}]	250
$D_{0,Fe^{3+}}$	Initial concentration of Fe^{3+} [mol m^{-3}]	250
E_{eq}	Equilibrium potential [V]	0.771 [9]
δ_m	Membrane thickness of FS-950 [m]	5×10^{-5}
R_m	Membrane resistance of FS-950 [Ω]	0.0392 (calculated)

6. Model validation of pressure drop

To verify the effectiveness of the model adopted in the present study, we compare the numerical results for pressure drop for the sponge-like NIPS electrodes to the experimental results. It can be found that the simulated pressure drop results are in good agreement with the experimental data. The discrepancy could stem from the intricate microstructure of the NIPS electrodes. This complexity might not be fully captured through macro-homogeneous parameter used in the model, such as assuming a uniform porosity. Additionally, the experimental pressure drop data exhibit higher non-linear deviations in the high electrolyte flow velocities, which could be attributed to the additional microscopic inertial effects. Note that the simulation analysis conducted in this study aims to qualitatively analyze the fluid dynamics. This approach facilitates a deeper understanding of the contributions of micro-patterned incorporations within the electrode architecture to enhancing performance. Precisely predicting electrochemical performance via an intricate pore-scale model exceeds the scope of the present study.

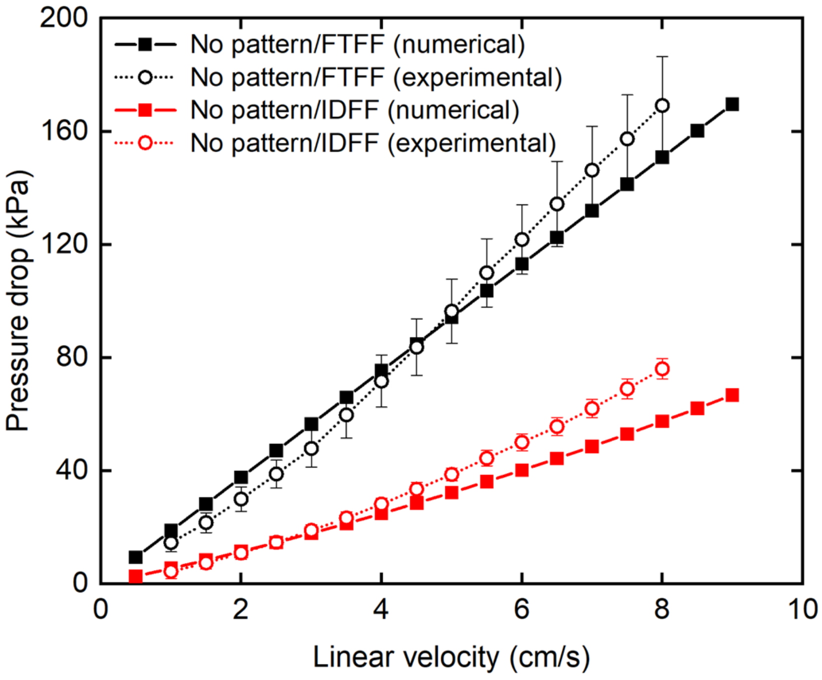

Figure S8. Pressure drop results obtained from both experiments and simulations for no pattern sponge-like NIPS electrodes in FTFF and IDFF configurations.

7. Electrode shrinkage effects after carbonization

In the present study, we observed that shrinkage during carbonization is not entirely uniform across the micro-patterned NIPS structures. Specifically, the linear shrinkage ratios after carbonization within the groove width and pillar diameter range from 20% to 25% (summarized in **Table S2**), while the bulk regions between adjacent grooves or pillars exhibit higher shrinkage of ~50% (also from the electrode thickness). For the groove-patterned electrodes, the groove depth is defined in the mold at 27.3% of the total electrode thickness prior to carbonization. After carbonization, the groove depth in both L-Groove and S-Groove designs remains at ~25-30% of the electrode thickness.

Table S2 Linear shrinkage ratios of micro-patterned electrodes after carbonization (N=6)

	L-Groove	S-Groove	L-Pillar	S-Pillar
Electrode thickness (μm)	520±15	486±12	526±8	529±8
Linear shrinkage ratio from electrode thickness	52.7±1.4%	55.8±1.1%	52.2±0.8%	51.9±0.8%
Linear shrinkage ratio from patterned width*	25.1±2.0%	23.2±3.4%	24.5±2.1%	24.2±3.7%

* This value is calculated based on linear dimensional changes of the groove widths and pillar diameters. All of the results are based on measurements from 6 batches of micro-patterned electrodes after carbonization, relative to the original geometric size of the molds.

8. Volume loss ratios for micro-patterned electrodes

Table S3 Calculated volume loss ratios from the micro-pattern designs

	L-Groove	S-Groove	L-Pillar	S-Pillar
Reduced volume ratio within one electrode*	9.1%	7.3%	9.3%	6.0%

* This value is calculated based on the original geometric size of the mold without consideration of electrode shrinkage effects after carbonization.

9. Permeability and non-linear Forchheimer coefficient of the NIPS electrodes

Table S4 Permeability and Forchheimer coefficient of sponge-like NIPS electrodes (n=2)

	Flow field	Permeability (κ)	Forchheimer coefficient (β)
No pattern	FTFF	$(1.0\pm0.1)\times10^{-11}$	$(7.8\pm0.9)\times10^{5}$
L-Groove	FTFF	$(1.6\pm0.4)\times10^{-11}$	$(3.0\pm0.3)\times10^{5}$
S-Groove	FTFF	$(2.3\pm0.4)\times10^{-11}$	$(3.6\pm0.4)\times10^{5}$
No pattern	IDFF	$(2.9\pm0.1)\times10^{-11}$	$(3.3\pm0.1)\times10^{5}$
L-Pillar	IDFF	$(5.7\pm0.1)\times10^{-11}$	$(3.9\pm0.2)\times10^{5}$
S-Pillar	IDFF	$(4.1\pm0.1)\times10^{-11}$	$(4.4\pm0.1)\times10^{5}$

Table S5 Permeability and Forchheimer coefficient of finger-shaped NIPS electrodes (n=2)

	Flow field	Permeability (κ)	Forchheimer coefficient (β)
No pattern	FTFF	$(2.7\pm0.3)\times10^{-11}$	$(8.4\pm0.6)\times10^{4}$
No pattern	IDFF	$(7.9\pm0.2)\times10^{-11}$	$(3.0\pm0.1)\times10^{5}$

10. Water contact angle measurements

Water contact angles were measured using a Krass DSA30S drop shape analyzer equipped with a CF04 camera and a high-intensity monochromatic LED light source. Sessile drop measurements were conducted on the top layer of the micro-patterned NIPS electrodes. For each measurement, a 5 μL deionized water droplet was dispensed onto the sample surface. Measurements were taken at three different locations per sample to account for surface variability. Apparent contact angles were determined using Krüss Advance software by applying a manual baseline and fitting the droplet profile with the Young-Laplace equation. The results show that all the three sponge-like NIPS electrodes exhibited similar apparent water contact angles of approximately 60°, indicating consistently hydrophilic surfaces.

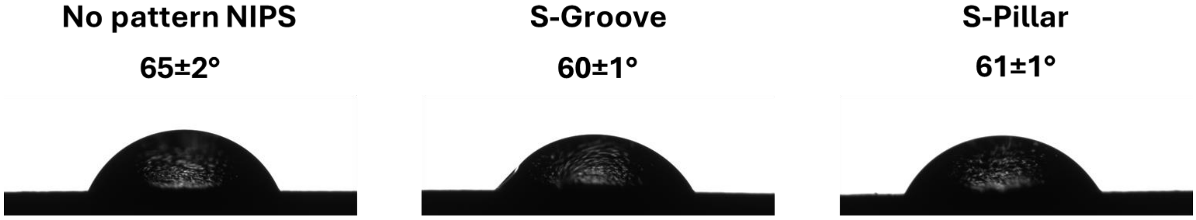

Figure S9. Contact angle measurements with ultrapure water as wetting liquid on the top surface of the micro-patterned sponge-like NIPS electrodes.

11. Mechanical compression tests

Mechanical stress-strain curves were measured using a Zwick/Roell Z 2.5 materials testing machine in compression mode at room temperature. Electrode samples were cut to a geometric area of ~70 mm^2 and placed between stainless steel plates. The moving crosshead was brought into contact with the electrode until a preload of 5 N was reached, which was defined as the zero-point for thickness (i.e., 0% compression ratio). Compression was then applied at a constant rate of 1 mm s^{-1}. The tests were continued until the electrode reached a maximum deformation of 80% relative to its original thickness. The results show that the patterned electrodes exhibit a similar, although slightly stiffer, compression response compared to the no pattern electrode. Under the 20% compression ratio used in the present study, all electrodes maintain good structural integrity, indicating their suitability for flow battery assembly. However, at a higher compression ratio of 40%, a reduction in stress is observed for the S-Pillar electrode, likely due to structural collapse within the pillar region. This suggests that excessive compression should be avoided in practical applications to preserve mechanical stability.

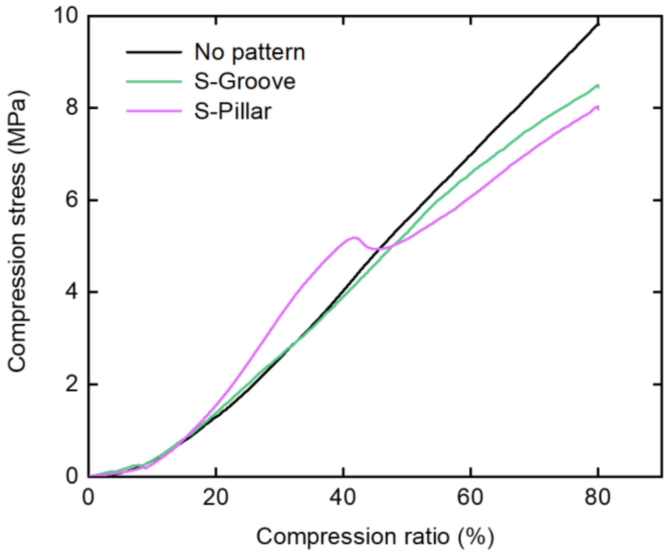

Figure S10 Strain-stress curves of the no pattern and micro-patterned sponge-like NIPS electrodes under the through-plane CRs of 0-80%.

12. Pressure drop measurements of the empty cell

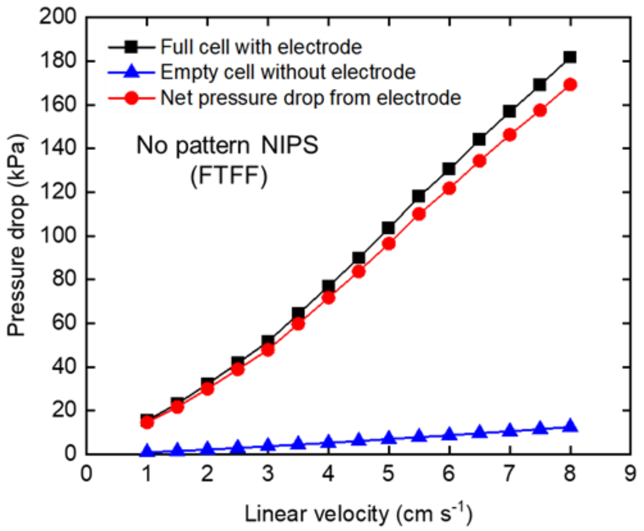

Figure S11. Pressure drop raw data for the empty cell (without electrode), full cell (with no-pattern NIPS electrode), and the calculated electrode-only contribution (by subtraction). Measurements were performed using the FTFF configuration.

13. Cyclic voltammetry measurements

As a complementary approach, cyclic voltammetry (CV) was performed in the single-electrolyte battery setup with 2 M HCl electrolyte solution, operating within a potential range of -0.2 V to 0.2 V without electrolyte flow, at scan rates of 20, 50, 100, 150, and 200 mV s^{-1} to estimate the electrochemical specific surface area (ECSA). Capacitance was calculated from $\bar{I} = C\frac{dV}{dt}$; where $\bar{I}$ represents the average of the positive and negative currents at 0 V, C is the capacitance, and dV/dt is the scan rate. The specific capacitance for glassy carbon (i.e., 18 mF cm^{-2}) was used for ECSA estimation. The cyclic voltammograms of the no pattern sponge-like NIPS electrode are shown in Fig. S9(a) and a linear fitting in Fig. S9(b). The volumetric ECSA is calculated as 48×10^6 m^2 m^{-3} (220 m^2 g^{-1}).

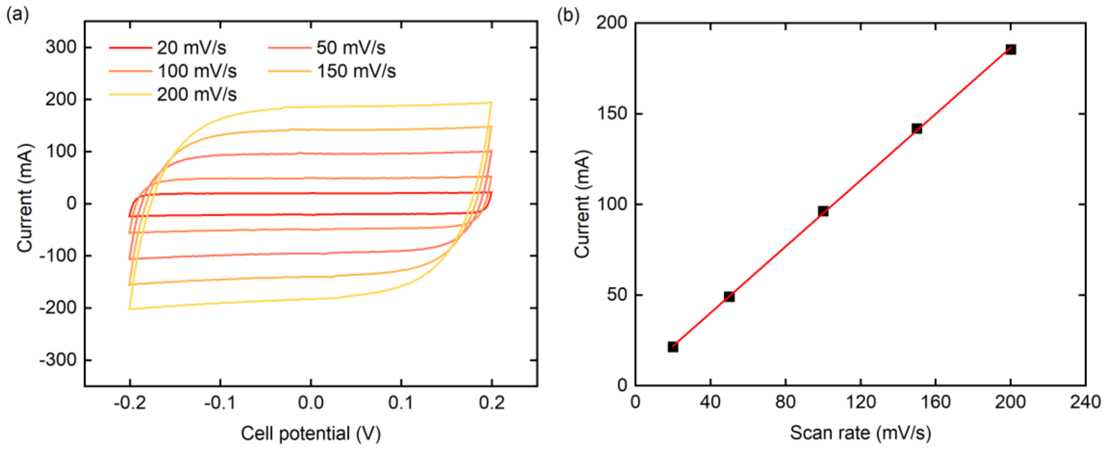

Figure S12. (a) Cyclic voltammograms of the no pattern sponge-like NIPS electrodes measured at the scan rates of 20, 50, 100, 150, and 200 mV s^{-1} in a flow cell with 2 M HCl as electrolyte. The electrolyte flow rate is set at 0 mL min^{-1}. (b) The averaged magnitudes of oxidative and reductive currents at 0 V, plotted as a function of scan rates ranging from 20 to 200 mV s^{-1}. A linear fitting is represented by the red line.

14. X-ray photoelectron spectroscopy (XPS) of electrode surface

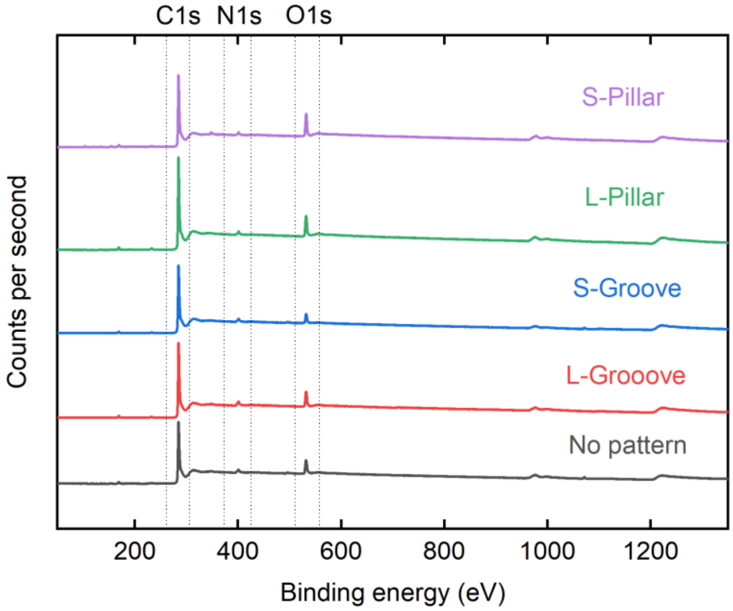

Figure S13. X-ray photoelectron spectroscopy (XPS) of the sponge-like NIPS electrodes. Measurements for all samples were taken at the top surface of the electrodes. The three main elemental composition (C1s, N1s, and O1s) are highlighted in the XPS spectra, and the other heteroatoms (e.g., Si, Fe, etc.) may come from the furnace during carbonization process.

Table S6 Summary of the elemental composition from XPS for sponge-like NIPS electrodes

	C1s (at.%)	N1s (at.%)	O1s (at.%)
No pattern	88.9	3.9	7.2
L-Groove	86.1	4.7	7.8
S-Groove	89.9	3.6	6.5
L-Pillar	87.1	2.6	8.8
S-Pillar	82.2	2.6	11.5

15. Ohmic resistances of NIPS electrodes extracted from EIS measurements

Table S7 Ohmic resistances of the NIPS electrodes extracted from the EIS measurements at high frequency regions in iron symmetric cell

	v_e ≈1 cm s^{-1}	v_e ≈2 cm s^{-1}	v_e ≈5 cm s^{-1}	v_e ≈10 cm s^{-1}
No pattern (FTFF)	0.22 Ω	0.21 Ω	0.22 Ω	-
L-Groove (FTFF)	0.25 Ω	0.25 Ω	0.26 Ω	0.26 Ω
S-Groove (FTFF)	0.27 Ω	0.27 Ω	0.26 Ω	0.26 Ω
No pattern (IDFF)	0.21 Ω	0.21 Ω	0.21 Ω	0.22 Ω
L-Pillar (IDFF)	0.21 Ω	0.22 Ω	0.21 Ω	0.21 Ω
S-Pillar (IDFF)	0.20 Ω	0.20 Ω	0.20 Ω	0.21 Ω

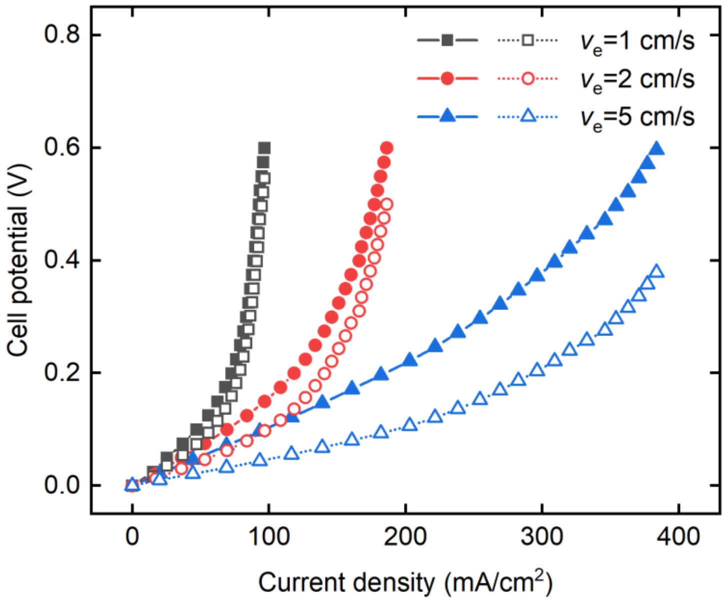

Figure S14. Polarization curves for the no pattern sponge-like NIPS electrodes, measured at different electrolyte flow velocities (v_e) under flow-through flow fields (FTFFs). Solid lines represent the full cell overpotentials, and dashed lines represent values after internal resistance (IR) correction.

16. Electrochemical performance of NIPS electrodes with micro-grooves

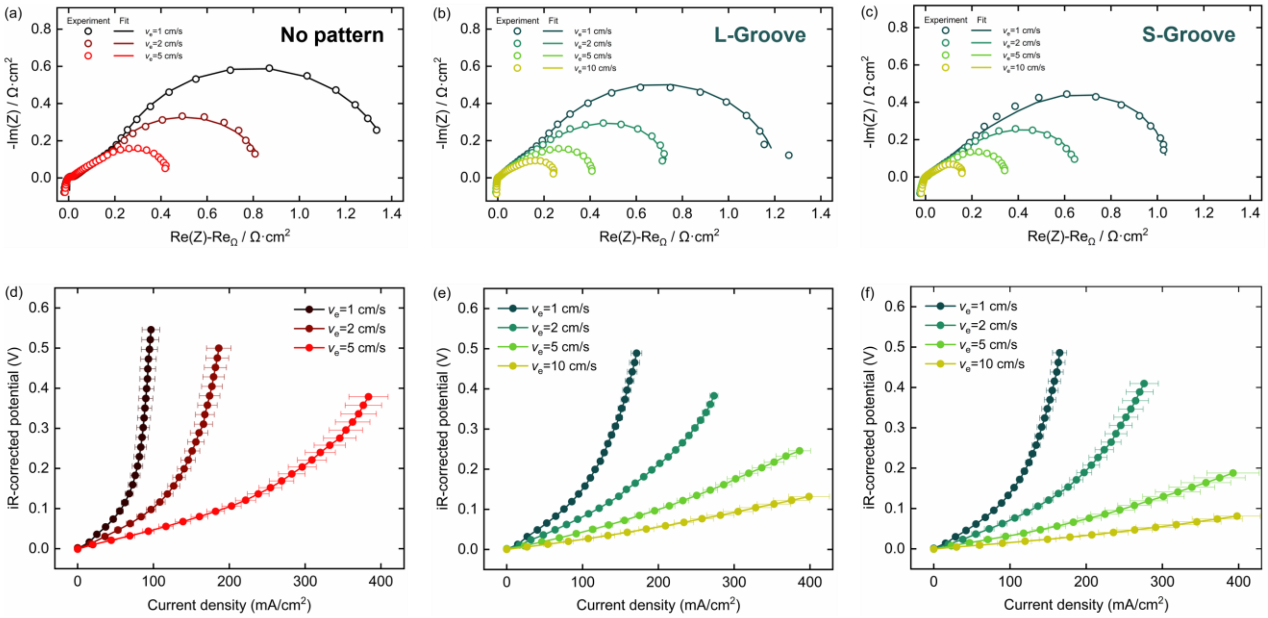

Figure S15 Electrochemical performance of the sponge-like NIPS electrodes with micro-grooves in a 0.5 M Fe^{2+}/Fe^{3+} symmetric cell under FTFFs. The performance of the no pattern NIPS electrodes is presented for comparison. Nyquist plots obtained through EIS measurements (dots) and the EIS fittings (lines) at different electrolyte linear velocities after subtracting the ohmic resistances are shown in (a)-(c). IR_Ω-corrected polarization curves are shown in (d)-(f). The error bars in polarization curves correspond to a standard deviation (n=2).

17. Pressure drop streamlines from cell continuum model

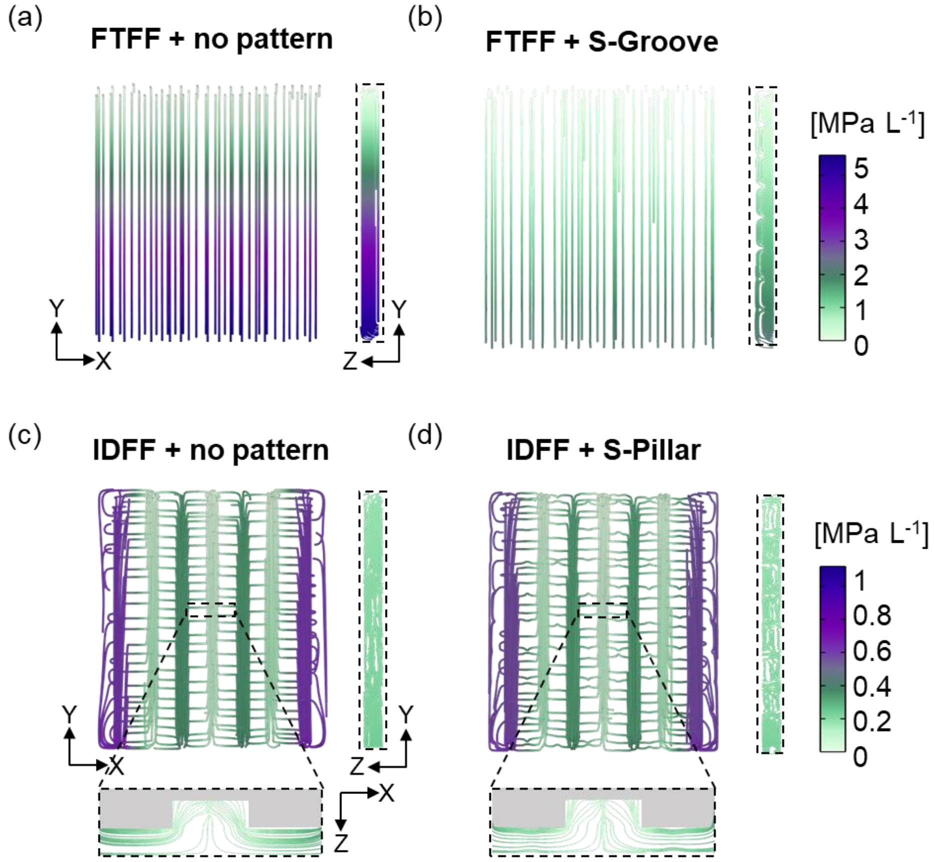

Figure S16 Normalized pressure drop streamlines per unit electrode length for NIPS electrodes at $v_e \approx 5$ cm s^{-1} using the continuum 3D half-cell model. (a) No pattern and (b) patterned electrodes (S-Groove) under FTFF, and (c) no pattern electrode and (d) patterned electrodes (S-Pillar) under IDFF.

18. Effects of the flow direction in the groove-patterned NIPS electrodes

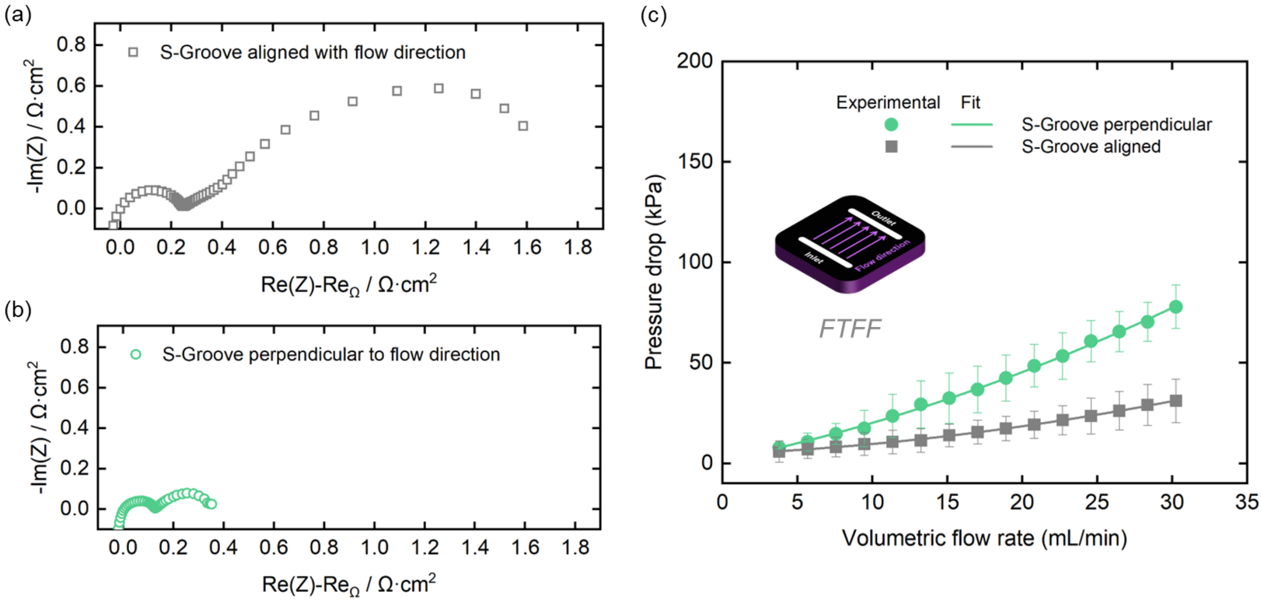

Figure S17 EIS spectra of the S-Groove NIPS electrodes with groove patterns (a) aligned with the flow direction and (b) perpendicular to the flow direction in a 1.6 M vanadium full cell at 50% SoC under FTFFs. The electrolyte velocity is selected as 5 cm s^{-1} for both designs. (c) Pressure drop measurements (dots) for both groove pattern orientations under FTFF in a single cell, along with the non-linear Darcy-Forchheimer fittings (lines). The error bars indicate standard deviation (n=2).

19. Electrochemical performance of NIPS electrodes with micro-pillars

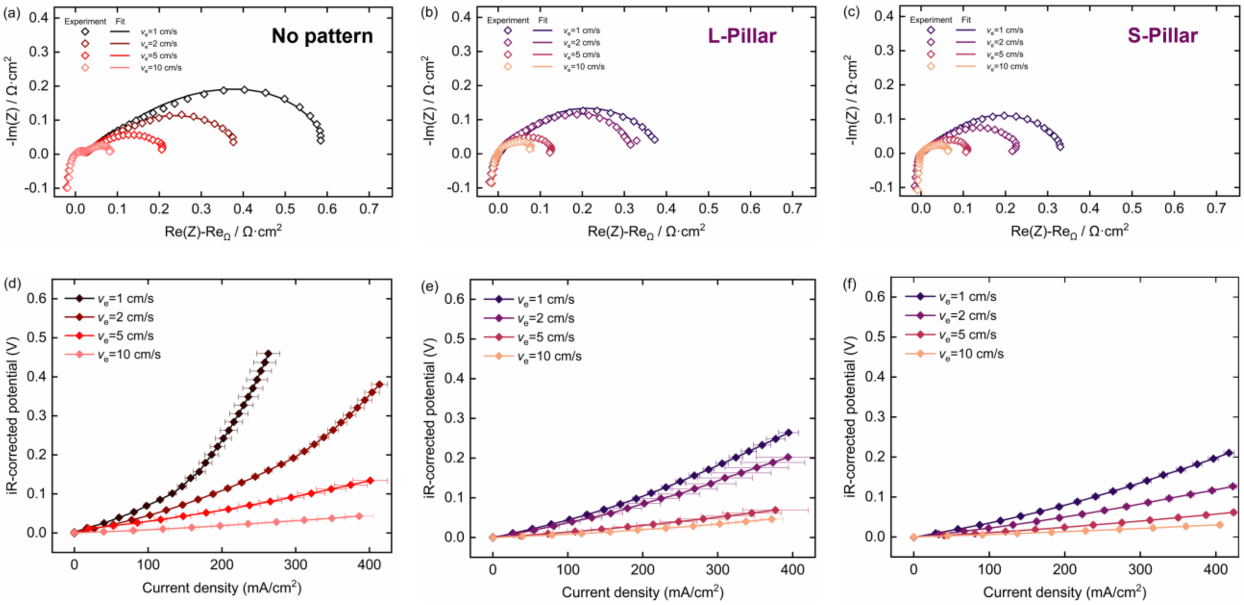

Figure S18 Electrochemical performance of the sponge-like NIPS electrodes with micro-pillars in a 0.5 M Fe^{2+}/Fe^{3+} symmetric cell under IDFFs. The performance of the no pattern NIPS electrodes is presented for comparison. Nyquist plots obtained through EIS measurements (dots) and the EIS fittings (lines) at different electrolyte linear velocities after subtracting the ohmic resistances are shown in (a)-(c). $IR_Ω$-corrected polarization curves are shown in (d)-(f). The error bars in polarization curves correspond to a standard deviation (n=2).

20. EIS fitting parameters

Table S8 EIS fitting parameters of the NIPS electrodes in a 0.5 M Fe^{2+}/Fe^{3+} symmetric cell under FTFF

Electrode type	v_e (cm s^{-1})	L (H)	R (Ω)	R_{CT} (Ω)	ρ_i (Ω cm^{-1})	Q_0 (S s^n cm^{-1})	q (unitless)	R_{MT} (Ω)
No pattern	1	1.2×10^{-7}	0.22	0.02	0.5	2.8×10^{-2}	0.85	0.54
L-Groove	1	1.2×10^{-7}	0.25	0.01	0.6	2.9×10^{-2}	0.94	0.47
S-Groove	1	1.4×10^{-7}	0.27	0.01	0.3	2.2×10^{-2}	0.83	0.39
No pattern	5	1.1×10^{-7}	0.22	0.01	0.4	2.4×10^{-2}	0.88	0.17
L-Groove	5	1.2×10^{-7}	0.26	0.01	0.2	2.7×10^{-2}	0.79	0.16
S-Groove	5	1.3×10^{-7}	0.26	0.01	0.2	1.5×10^{-2}	0.82	0.13

Table S9 EIS fitting parameters of the NIPS electrodes in a 0.5 M Fe^{2+}/Fe^{3+} symmetric cell under IDFF

Electrode type	v_e (cm s^{-1})	L (H)	R (Ω)	R_{CT} (Ω)	ρ_i (Ω cm^{-1})	Q_0 (S s^n cm^{-1})	q (unitless)	R_{MT} (Ω)
No pattern	1	4.6×10^{-8}	0.21	0.01	0.5	1.3×10^{-2}	0.79	0.22
L-Pillar	1	1.3×10^{-7}	0.21	0.01	0.3	1.3×10^{-2}	0.81	0.14
S-Pillar	1	1.1×10^{-7}	0.20	0.01	0.2	1.1×10^{-2}	0.76	0.12
No pattern	5	5.8×10^{-8}	0.21	0.01	0.5	3.3×10^{-2}	0.78	0.07
L-Pillar	5	1.4×10^{-7}	0.21	0.01	0.3	2.4×10^{-2}	0.68	0.05
S-Pillar	5	1.3×10^{-7}	0.20	0.01	0.1	2.1×10^{-2}	0.74	0.03

21. Comparison with commercial carbon-fiber electrodes

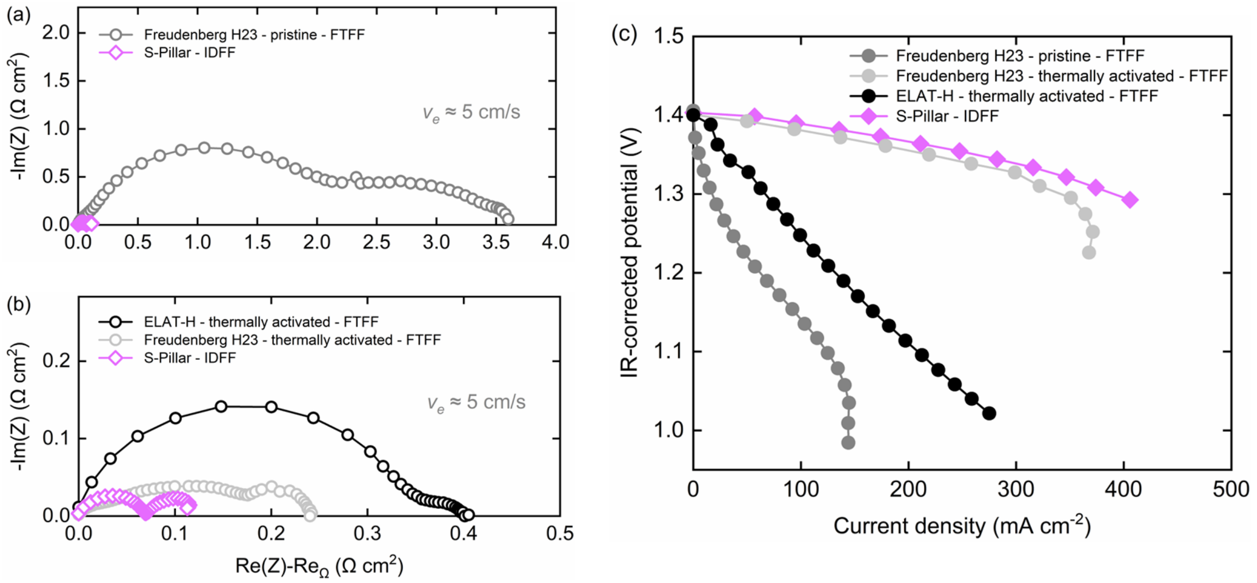

Figure S19 Electrochemical performance comparison of the S-Pillar NIPS electrodes with commercial carbon-fiber electrodes. (a)-(b) EIS spectra and (c) discharge polarization curve in a 1.6 M full-cell vanadium RFB at 50% SoC, measured at $v_e \approx 5$ cm s^{-1}.

22. References

[1] R. R. Jacquemond, M. van der Heijden, E. B. Boz, E. R. C. Ruiz, K. V. Greco, J. A. Kowalski, V. Muñoz-Perales, F. R. Brushett, K. Nijmeijer, P. Boillat, A. Forner-Cuenca, *Nat. Commun.* **2024**, *15*, 7434.
DOI: 10.1038/s41467-024-50120-7

[2] V. Muñoz-Perales, M. van der Heijden, P. A. García-Salaberri, M. Vera, A. Forner-Cuenca, *ACS Sustainable Chem. Eng*. **2023**, *11*, 12243.
DOI: 10.1021/acssuschemeng.3c00848.

[3] J. D. Milshtein, K. M. Tenny, J. L. Barton, J. Drake, R. M. Darling, F. R. Brushett, *J. Electrochem. Soc*. **2017**, *164*, E3265.
DOI: 10.1149/2.0201711jes.

[4] E. B. Boz, P. Boillat, A. Forner-Cuenca, *ACS Appl. Mater. Interfaces* **2022**, *14*, 41883.
DOI: 10.1021/acsami.2c08211.

[5] Z. Galus, R. N. Adams, *J. Phys. Chem.* **1963**, 1963, *67*, 866.
DOI: 10.1021/j100798a036.

[6] M. van der Heijden, R. van Gorp, M. A. Sadeghi, J. Gostick, A. Forner-Cuenca, *J. Electrochem. Soc.* **2022**, *169*, 040505.
DOI: 10.1149/1945-7111/ac5e46.

[7] E. Nishikata, T. Ishii, y T. Ohta, *J. Chem. Eng. Data* **1981**, *26*, 254.
DOI: 10.1021/je00025a008.

[8] K. L. Hawthorne, J. S. Wainright, R. F. Savinell, *J. Electrochem. Soc.* **2014**, *161*, A1662.
DOI: 10.1149/2.0761410jes.

[9] A. Z. Weber, M. M. Mench, J. P. Meyers, P. N. Ross, J. T. Gostick, Q. Liu, *J. Appl. Electrochem.* **2011**, *41*, 1137.
DOI: 10.1007/s10800-011-0348-2.
